# Total synthesis of clostrubin

**DOI:** 10.1038/ncomms7445

**Published:** 2015-03-11

**Authors:** Ming Yang, Jian Li, Ang Li

**Affiliations:** 1 State Key Laboratory of Bioorganic and Natural Products Chemistry, Collaborative Innovation Center of Chemistry for Life Sciences, Shanghai Institute of Organic Chemistry, Chinese Academy of Sciences, 345 Lingling Road, Shanghai 200032, China

## Abstract

Clostrubin is a potent antibiotic against methicillin- and vancomycin-resistant bacteria that was isolated from a strictly anaerobic bacterium *Clostridium beijerinckii* in 2014. This polyphenol possesses a fully substituted arene moiety on its pentacyclic scaffold, which poses a considerable challenge for chemical synthesis. Here we report the first total synthesis of clostrubin in nine steps (the longest linear sequence). A desymmetrization strategy is exploited based on the inherent structural feature of the natural product. Barton–Kellogg olefination forges the two segments together to form a tetrasubstituted alkene. A photo-induced 6π electrocyclization followed by spontaneous aromatization constructs the hexasubstituted B ring at a late stage. In total, 200 mg of clostrubin are delivered through this approach.

## Abstract (web summary)

Due to rising resistance, efficient routes to new antibiotics is a vital task for human health. Here, the authors report a short, convergent and elegant synthesis of a very recently reported antibiotic, successfully giving access to this material on scale.

## Graphical abstract

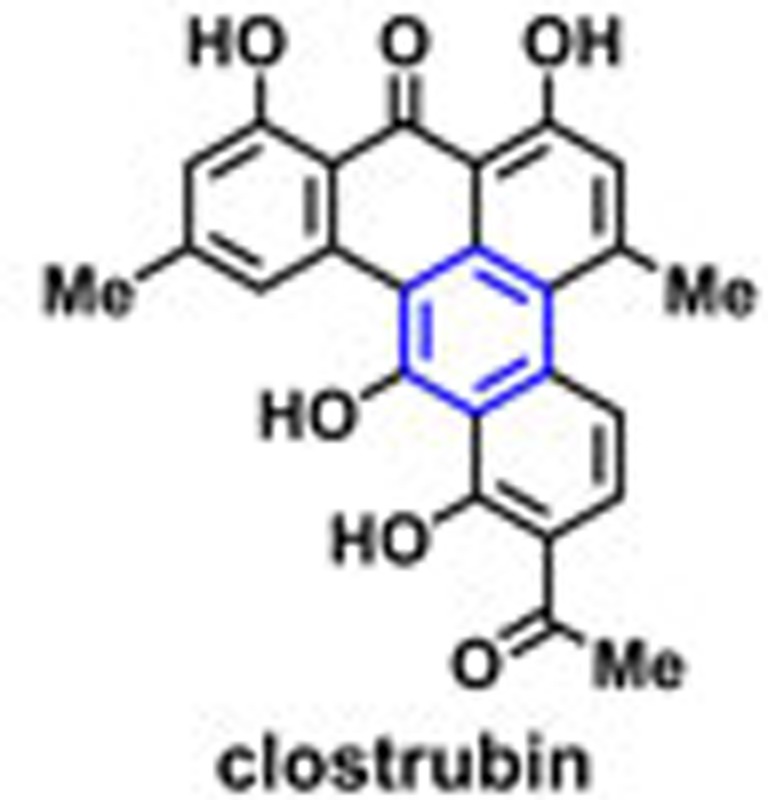

The discovery of effective antibiotic agents is an urgent global demand for combating drug-resistant pathogenic bacterium strains^1^,^2^. Secondary metabolites that are produced by microbes as chemical defence have proven to be the most important source of such agents^3^,^4^,^5^,^6^,^7^,^8^,^9^,^10^,^11^,^12^,^13^,^14^. In May 2014, Hertweck and co-workers^15^ reported the isolation of clostrubin (**1**, Fig. 1) from the strictly anaerobic bacterium *C. beijerinckii*. This compound exhibits remarkable potency against methicillin-resistant *Staphylococcus aureus* and vancomycin-resistant enterocci, with minimum inhibitory concentrations of 0.12 and 0.97 μM, respectively. From a structural perspective, clostrubin (**1**) poses considerable synthetic challenge owing to the fused aromatic ring system and multisubstitution pattern. The potential of **1** as a lead compound for antibiotic development and its limited supply from natural sources stimulated us to launch a chemical synthesis programme immediately.

In this paper, we report the first total synthesis of clostrubin (**1**) from commercially available starting materials. This concise synthesis (nine-step for the longest linear sequence) benefits from the inherent structural symmetry of **1**. An advanced olefin intermediate was constructed through Barton–Kellogg olefination, and a 6π electrocyclization promoted by ultraviolet light assembled the fully substituted B ring.

## Results

### Retrosynthetic analysis

**Figure 1:**
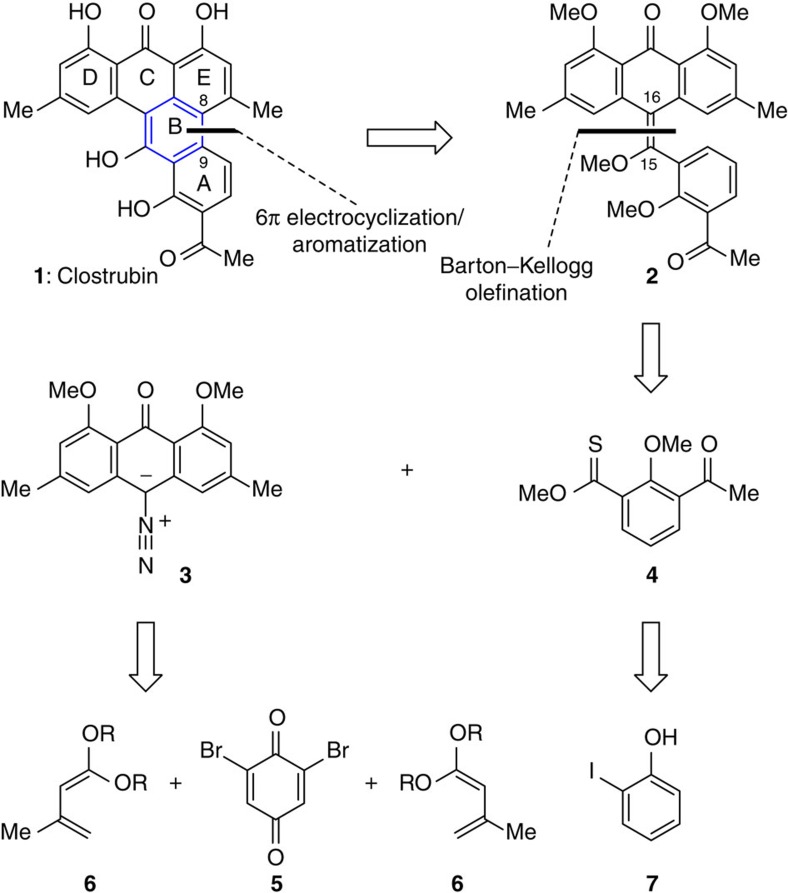
Retrosynthetic analysis of clostrubin. The inherent structural symmetry of the molecule inspires a desymmetrization strategy. A Barton–Kellogg olefination would be used for assembling a sterically hindered tetrasubstituted olefin. A 6π electrocyclization/aromatization sequence is envisioned as the key step for the construction of the hexasubstituted aromatic B ring.

We undertook a retrosynthetic analysis of clostrubin (**1**) taking advantage of the inherent symmetry^16^ of its molecular architecture, as illustrated in Fig. 1. The initial disassembly of the fully substituted B ring leads to a precursor **2**; the recombination of the sterically hindered C8–C9 biaryl bond could rely on a 6π electrocyclization reaction. Electrocyclization has emerged as a powerful tool for the construction of fused ring systems since Nicolaou’s pioneering synthesis of endiandric acids^17^,^18^,^19^,^20^,^21^,^22^,^23^,^24^,^25^,^26^,^27^,^28^,^29^,^30^,^31^,^32^. In particular, the strategy of 6π electrocyclization/aromatization has demonstrated a significant advantage in the synthesis of multisubstituted arenes^33^,^34^,^35^,^36^,^37^,^38^,^39^,^40^,^41^,^42^,^43^,^44^. Recently, we exploited such a strategy in the total syntheses of a series of natural products, such as tubingensin A, daphenylline, xiamycin and oridamycin families, and rubriflordilactone A^45^,^46^,^47^,^48^. It should be mentioned that the construction of hexasubstituted arenes using such strategy is rare in natural product synthesis. Preparing the symmetrical, tetrasubstituted olefin **2** is indeed of significant challenge with the conventional olefination methods, and Barton–Kellogg reaction is envisioned as a suitable solution^49^,^50^,^51^,^52^,^53^,^54^. Interestingly, this olefination method has found remarkable applications in material science^55^,^56^,^57^ rather than natural product synthesis in recent years. Disconnection at the C15–C16 bond results in stabilized diazo compound **3** and thioester **4**. The former may arise from the corresponding anthraquinone precursor, which is further traced back to 2,6-dibromo-1,4-benzoquinone (ref. 58) **5** and Brassard diene (refs 59, 60) **6** in a double retro-Diels–Alder (D-A) manner. The latter would be accessed from commercially available iodide **7** by using lithium chemistry.

### Synthesis of the two segments

**Figure 2:**
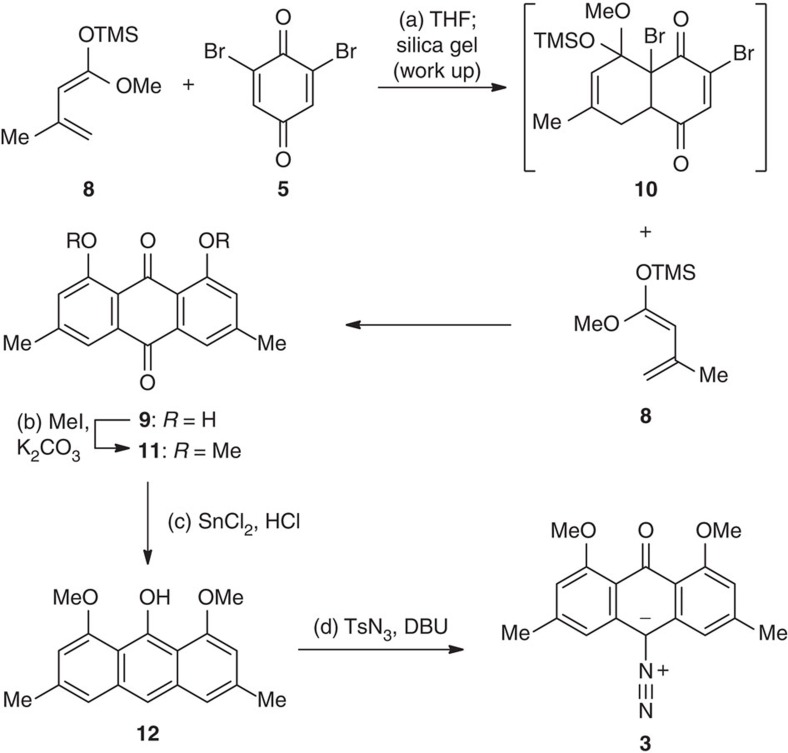
Synthesis of the anthraquinone segment. Reagents and conditions: (a) tetrahydrofuran (THF), −78 °C, 6 h; then silica gel, 22 °C, 2 h. (b) K_2_CO_3_ (3.0 eq), MeI (2.5 eq), *N*,*N*-dimethylformamide (DMF), 22 °C, 18 h, 45% (two steps). (c) SnCl_2_·H_2_O (6.0 eq), aqueous HCl (37 wt%), AcOH, 22 °C, 30 min. (d) DBU (3.0 eq), TsN_3_ (1.1 eq), methylene chloride (CH_2_Cl_2_), 22 °C, 20 h, 89% (two steps). TMS, trimethylsilyl.

The synthesis commenced with the preparation of diazoketone **3**, as shown in Fig. 2. A sequence of double D-A reactions was carried out: 2,6-dibromo-1,4-benzoquinone **5**, prepared in one step from commercially available 1,3,5-tribromophenol^58^, reacted smoothly with an excess of diene **8** to afford anthraquinone **9** along with a small amount of mono-*O*-methyl-**9**, presumably via the intermediacy of **10**. The regioselectivity of the D-A reactions may be attributable to the electronic bias induced by the bromine atom. The addition of silica gel may accelerate the hydrolysis of the silyl ether and thus led to a rapid aromatization along with release of HBr. Notably, when a single equivalent of **8** was used for the D-A reaction, a bromonaphthoquinone was readily prepared, presumably with the intermediacy of a mono cycloadduct^61^. The crude **9** was treated with K_2_CO_3_ and MeI to give compound **11** (45% overall yield from **5**). **11** underwent Clemmensen-type reduction in the presence of SnCl_2_ and HCl to give the corresponding monoketone^62^, which instantaneously tautomerized to anthranol **12**. Exposure of crude **12** to 1,8-diazabicyclo[5.4.0]undec-7-ene (DBU) and TsN_3_ furnished diazoketone **3** in 89% overall yield.

**Figure 3:**
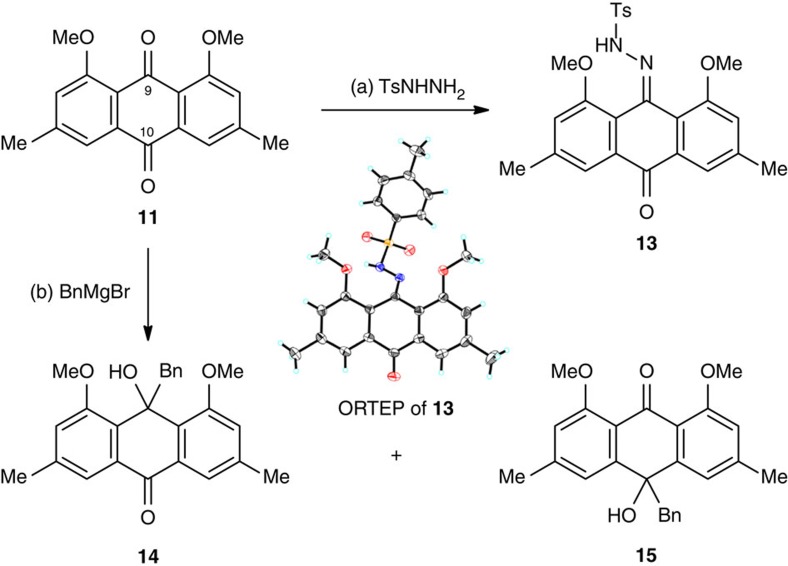
**Unusual reactivity** **of the anthraquinone intermediate.** Reagents and conditions: (a) TsNHNH_2_ (1.0 eq), TsOH·H_2_O (10 mol%), EtOH, 60 °C, 2 h, 61%. (b) BnMgBr (1.0 eq), tetrahydrofuran (THF), 22 °C, 15 min, 32% for **14** and 40% for **15**. ORTEP, Oak Ridge Thermal Ellipsoid Plot.

We observed unexpected reactivity of anthraquinone **11** (Fig. 3) during the above studies, which influenced the overall strategy of the synthesis. In theory, the C9 carbonyl of **11** should be sterically more hindered for nucleophilic attack due to two neighbouring methoxy groups. From an electronic perspective, this upper ketone could be considered as an equivalent of a double vinylogous carbonate than is also rather unreactive as an electrophile. To our surprise, we obtained hydrazone **13** in 61% yield when treating **11** with TsNHNH_2_; the anticipated regioisomer was not detected. The structure of **13** was determined by X-ray crystallographic analysis. This observation interrupted our initial plan of exploiting the C10 tosylhydrazone of **11** as the potential precursor for the Barton–Kellogg olefination. We further examined other types of nucleophiles such as benzyl Grignard reagent for the addition reaction with **11**. In this case, two regioisomeric alcohols **14** and **15** were isolated in 32% and 40% yields, respectively. Both structures were confirmed by nuclear Overhauser effect (NOE) studies. The enhanced reactivity of the C9 carbonyl may be attributable to inductive effects from the *o*-methoxy substitutents or relief of 1,3-allylic strain that occurs on nucleophilic additions. Thus, the strategy involving direct olefination of C10 carbonyl of anthraquinone **11** (for example, with functionalized benzylic metal species or phosphonate carbanion) had to be abandoned due to the poor regioselectivity.

**Figure 4:**
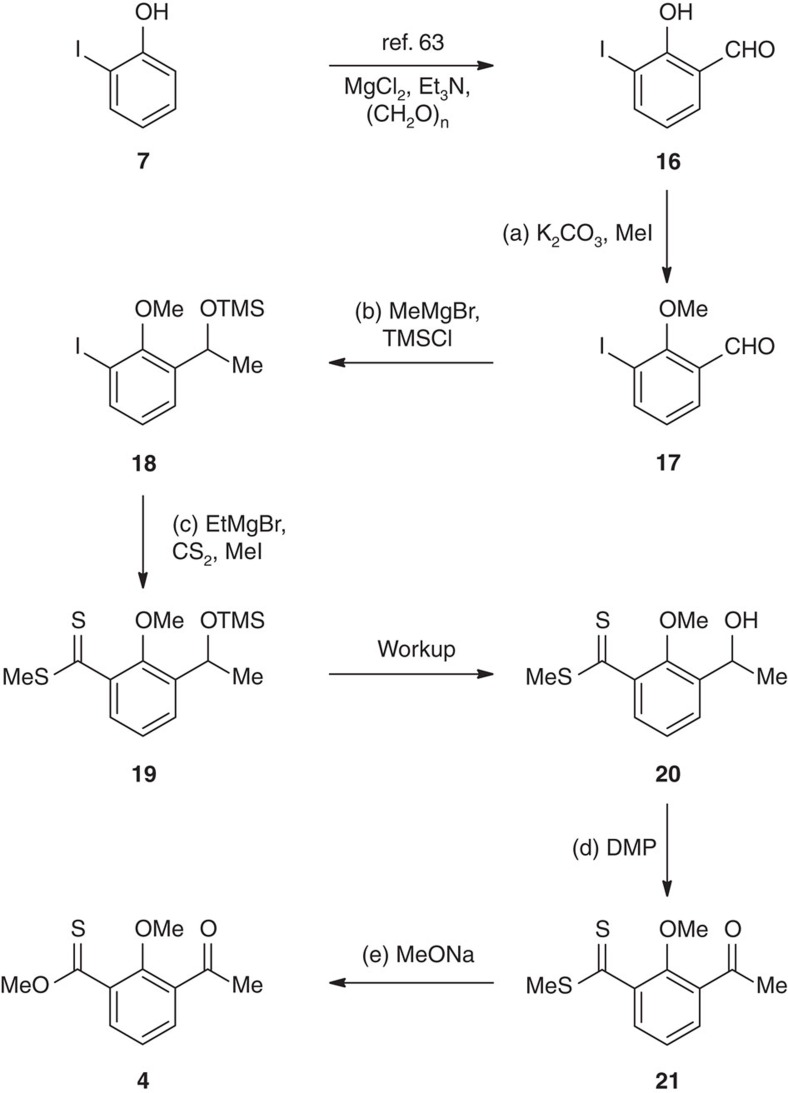
Synthesis of the thioester segment. Reagents and conditions: (a) K_2_CO_3_ (1.5 eq), MeI (1.5 eq), *N*,*N*-dimethylformamide (DMF), 22 °C, 20 h, 99%. (b) MeMgBr (1.05 eq), 0 °C, 10 min; then HMPA (2.0 eq), triethylamine (Et_3_N; 1.5 eq), chlorotrimethylsilane (TMSCl; 1.5 eq), tetrahydrofuran (THF), 22 °C, 10 min, 94%. (c) EtMgBr (1.5 eq), THF, 50 °C, 1 h; then CS_2_ (30 eq), 70 °C, 3 h; then MeI (4.0 eq), 22 °C, 8 h, 67%. (d) DMP (1.2 eq), methylene chloride (CH_2_Cl_2_), 22 °C, 1 min, 83%. (e) MeONa (10.0 eq), methanol (MeOH), 50 °C, 4 h, 66%.

We then focused on the synthesis of the thioester segment as the electron donor in the devised Barton–Kellogg olefination, as shown in Fig. 4. Aldehyde **16** was prepared in one step from commercially available 2-iodophenol^63^. Treatment with K_2_CO_3_ and MeI gave methyl ether **17** (99% yield), which underwent MeMgBr addition followed by silylation to provide compound **18** (94% yield) in one pot. Hexamethylphosphoramide (HMPA) was found to be crucial to enhance the nucleophilicity of the magnesium alkoxide. **18** was subjected to the magnesium–halogen exchange conditions (EtMgBr) to generate a functionalized Grignard reagent^45^,^47^,^64^,^65^,^66^, which was quenched by CS_2_ and MeI to give dithioester **19**. It is noteworthy that lithium–halogen exchange did not lead to a satisfactory result. The desilylation took place spontaneously during acid workup to deliver alcohol **20** in 67% yield from **18**. Oxidation of **20** with Dess–Martin periodinane (DMP) afforded ketone **21** (83% yield) without destroying the sulfur-containing functionalities, and the subsequent methanolysis furnished thioester **4** in 66% yield.

### Completion of the synthesis

**Figure 5:**
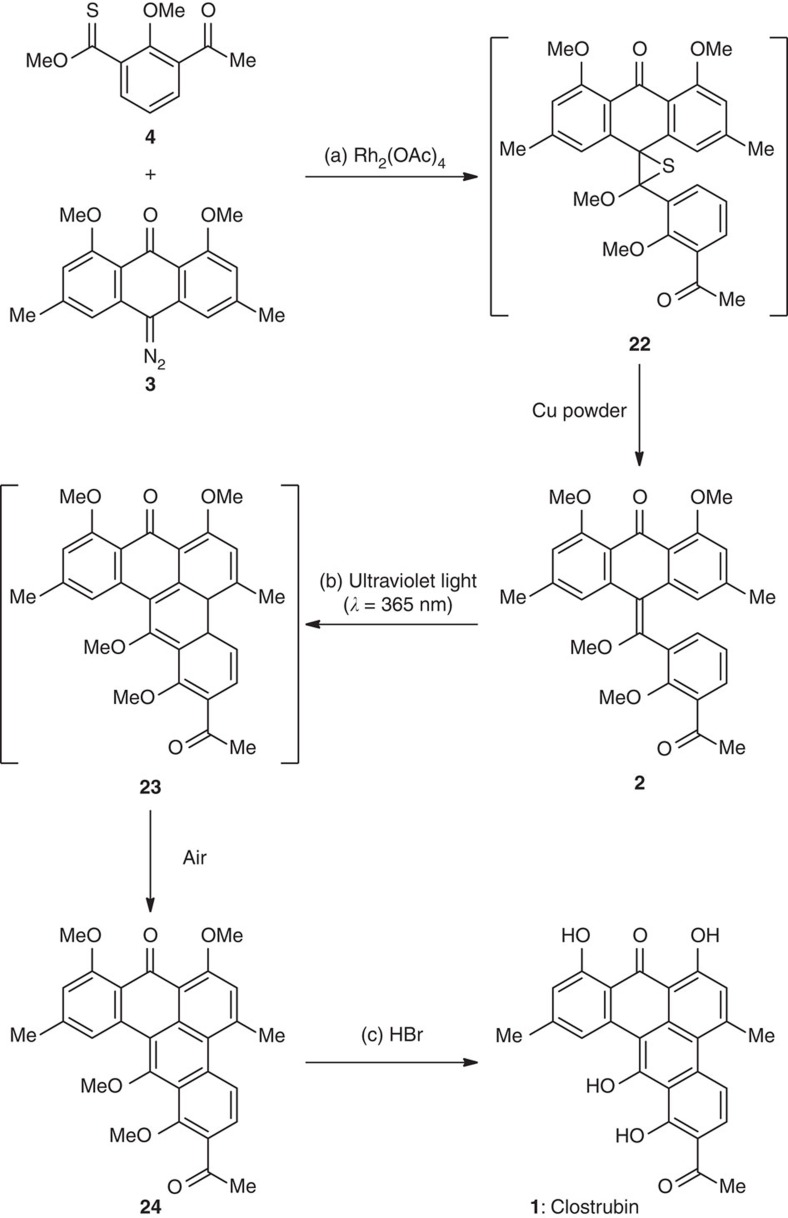
Completion of the synthesis of clostrubin. Reagents and conditions: (a) Rh_2_(OAc)_4_ (5 mol%), toluene, 50 °C, 55 min; then Cu powder, 110 °C, 1.5 h, 85%. (b) ultraviolet light (*λ*=365 nm, Hg lamp), CHCl_3_, 22 °C, 8 h, 55%. (c) aq. HBr (48 wt%), AcOH, 120 °C, 10 h, 95%.

With both fragments in hand, we directed our attention to the construction of the aromatic B ring, as depicted in Fig. 5. It is well documented in the literature of Barton–Kellogg olefination that thioketones readily react with diazo compounds without promoters or catalysts^55^,^56^,^57^. After examination of the conventional conditions, we realized that the stabilized diazoketone **3** needed to be activated by Rh_2_(OAc)_4_ to form the metal-carbenoid intermediate^52^,^54^,^67^,^68^,^69^,^70^, which was further trapped by relatively unreactive thioester **4**. The postulated episulfide intermediate **22** was reduced by Cu powder *in situ* to afford tetrasubstituted olefin **2** in 85% overall yield. We examined a series of conditions such as heating or FeCl_3_ to promote the last C–C bond formation but only observed decomposition. Inspired by our synthesis of daphenylline^46^, we irradiated **2** with ultraviolet light (*λ*=365 nm). To our delight, this symmetrical olefin underwent a 6π electrocyclization, presumably to provide pentacyclic intermediate **23**, which was spontaneously oxidized under an air atmosphere during workup to furnish tetramethyl clostrubin **24** (55% yield from **2**). Global deprotection of the methyl groups with aqueous HBr (48 wt%) gave clostrubin (**1**) with excellent efficiency. The spectra and physical properties of the synthetic **1** are consistent with those reported for the natural product. In total, 200 mg of **1** were obtained through the synthesis.

## Discussion

In summary, we have accomplished the first total synthesis of clostrubin. The concise and efficient route took advantage of the 6π electrocyclization strategy as well as the inherent structural symmetry of the molecule. The synthesis provides a practical means to obtain this potent antibiotic for further investigations, considering the limited supply and difficult isolation of the naturally occurring sample.

## Methods

### General

All reactions were carried out under an argon atmosphere with dry solvents under anhydrous conditions, unless otherwise noted. Tetrahydrofuran was distilled immediately before use from sodium-benzophenone ketyl. Methylene chloride, *N*,*N*-dimethylformamide, triethylamine, *N*,*N*-diisopropylethylamine and chlorotrimethylsilane were distilled from calcium hydride and stored under an argon atmosphere. Methanol was distilled from magnesium and stored under an argon atmosphere. Reagents were purchased at the highest commercial quality and used without further purification, unless otherwise stated. Solvents for chromatography were used as supplied by Titan Chemical. Reactions were monitored by thin-layer chromatography carried out on S-2 0.25 mm E. Merck silica gel plates (60F-254) using ultraviolet light as visualizing agent and aqueous ammonium cerium nitrate/ammonium molybdate or basic aqueous potassium permanganate as developing agent. E. Merck silica gel (60, particle size 0.040–0.063 mm) was used for flash column chromatography. Preparative thin-layer chromatography separations were carried out on 0.25 or 0.50 mm E. Merck silica gel plates (60F-254). Nuclear magnetic resonance (NMR) spectra were recorded on Bruker AV-400 or Agilent 500/54/ASP instrument and calibrated by using residual undeuterated chloroform (*δ*_H_=7.26 p.p.m.) and CDCl_3_ (*δ*_C_=77.16 p.p.m.) or undeuterated dimethylsulfoxide (*δ*_H_=2.50 p.p.m.) and dimethylsulfoxide-d_6_ (*δ*_C_=39.52 p.p.m.), as internal references. Infrared spectra were recorded on a Thermo Scientific Nicolet 380 FT-IR spectrometer. Melting points are uncorrected and were recorded on a Shanghai Jingke SGW X-4 apparatus. High-resolution mass spectra were recorded on a Bruker APEXIII 7.0 Tesla ESI-FT or a Waters Micromass GCT Premier EI mass spectrometer.

For ^1^H and ^13^C NMR spectra of compounds, see Supplementary Figs 1–26. For heteronuclear multiple quantum correlation spectroscopy (HMQC) and heteronuclear multiple-bond correlation spectroscopy (HMBC) spectra of compound **13**, see Supplementary Figs 27 and 28. For nuclear Overhauser effect spectroscopy (NOESY) spectra of compound **14** and **15**, see Supplementary Figs 29 and 30. For the comparisons of ^1^H and ^13^C NMR spectra of the natural and synthetic clostrubin, see Supplementary Figs 31 and 32. For the comparisons of ^1^H and ^13^C NMR spectroscopic data of the natural and synthetic clostrubin, see Supplementary Tables 1 and 2. For the experimental procedures and spectroscopic and physical data of compounds and the crystallographic data of compound **13**, see Supplementary Methods.

## Author contributions

A.L., M.Y. and J.L. conceived the synthetic route; A.L. directed the project; M.Y. and J.L. conducted the work; A.L., M.Y. and J.L. analysed the results; and A.L. wrote the manuscript.

## Additional information

**Accession codes:** The X-ray crystallographic coordinates for structure (**13**) reported in this article have been deposited at the Cambridge Crystallographic Data Centre (CCDC), under deposition number CCDC 1028256. These data can be obtained free of charge from The Cambridge Crystallographic Data Centre via www.ccdc.cam.ac.uk/data_request/cif.

**How to cite this article:** Yang, M. *et al*. Total synthesis of clostrubin. *Nat. Commun.* 6:6445 doi: 10.1038/ncomms7445 (2015).

## Supplementary Material

Supplementary Information Supplementary Figures 1-32, Supplementary Table 1-2, Supplementary Methods, Supplementary References
