# Supplementary material for: Total synthesis of clostrubin
Source: Nat Commun. 2015 Mar 11;6:6445. doi: 10.1038/ncomms7445 (PMC4382683; doi:10.1038/ncomms7445)
Supplement: Supplementary Information — Supplementary Figures 1-32, Supplementary Table 1-2, Supplementary Methods, Supplementary References [file ncomms7445-s1.pdf]

Supplementary Figure 1. ^{1}H NMR Spectrum of 11 (500 MHz, $CDCl_3$)

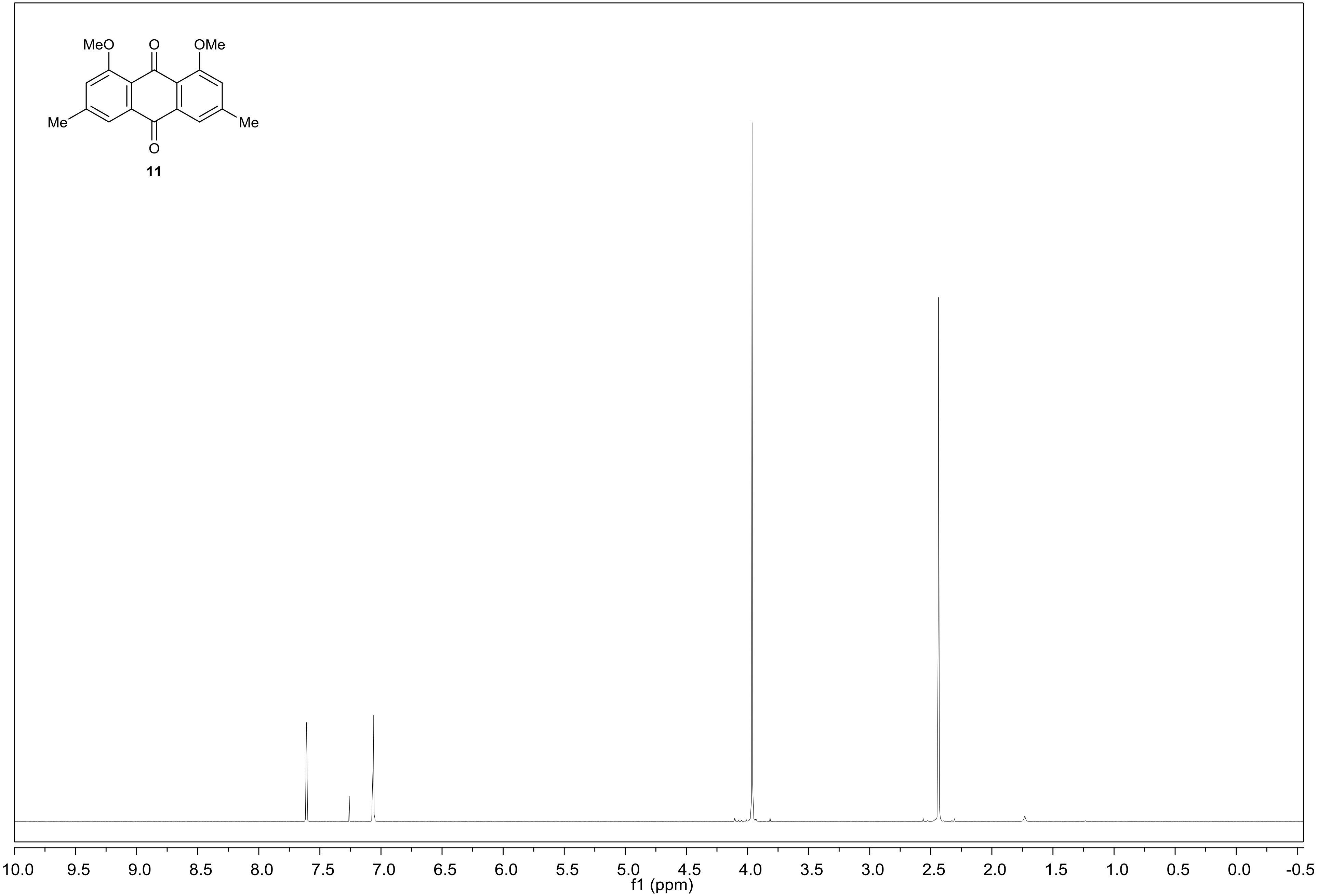

Supplementary Figure 2. ^{13}C NMR Spectrum of 11 (126 MHz, $CDCl_3$)

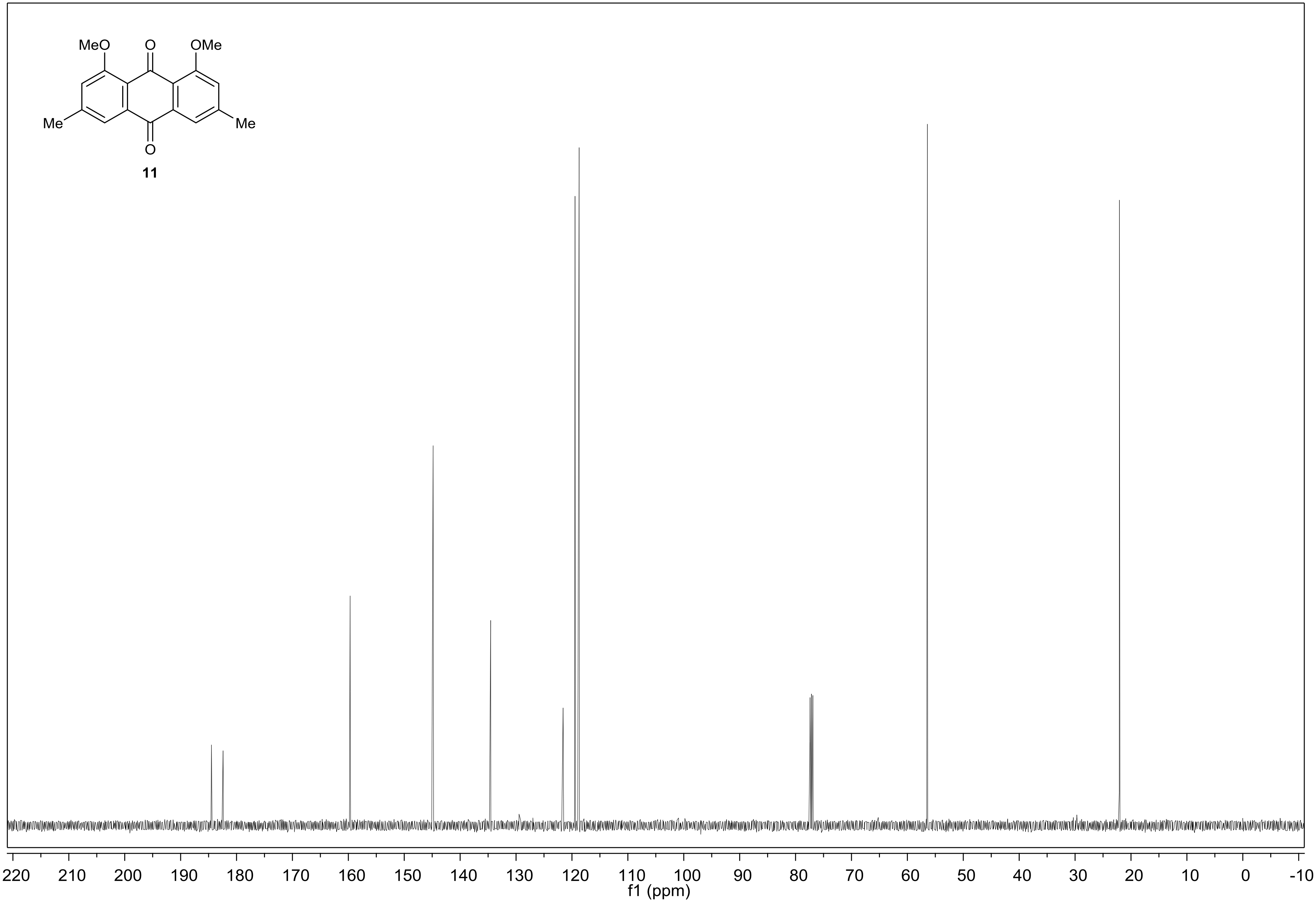

Supplementary Figure 3. ^{1}H NMR Spectrum of 3 (500 MHz, $CDCl_3$)

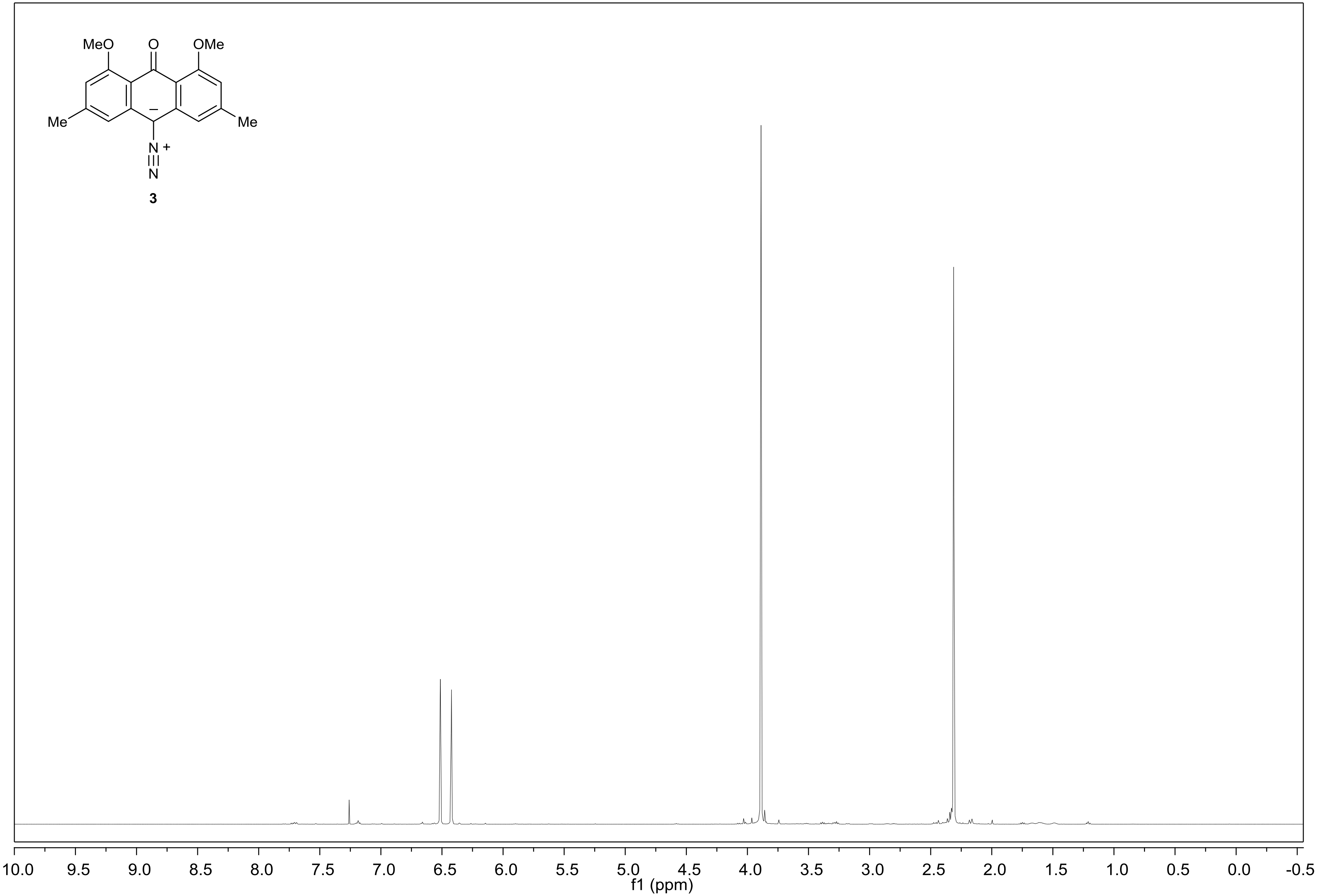

Supplementary Figure 4. ^{13}C **NMR Spectrum of 3 (126 MHz,** $CDCl_3$**)**

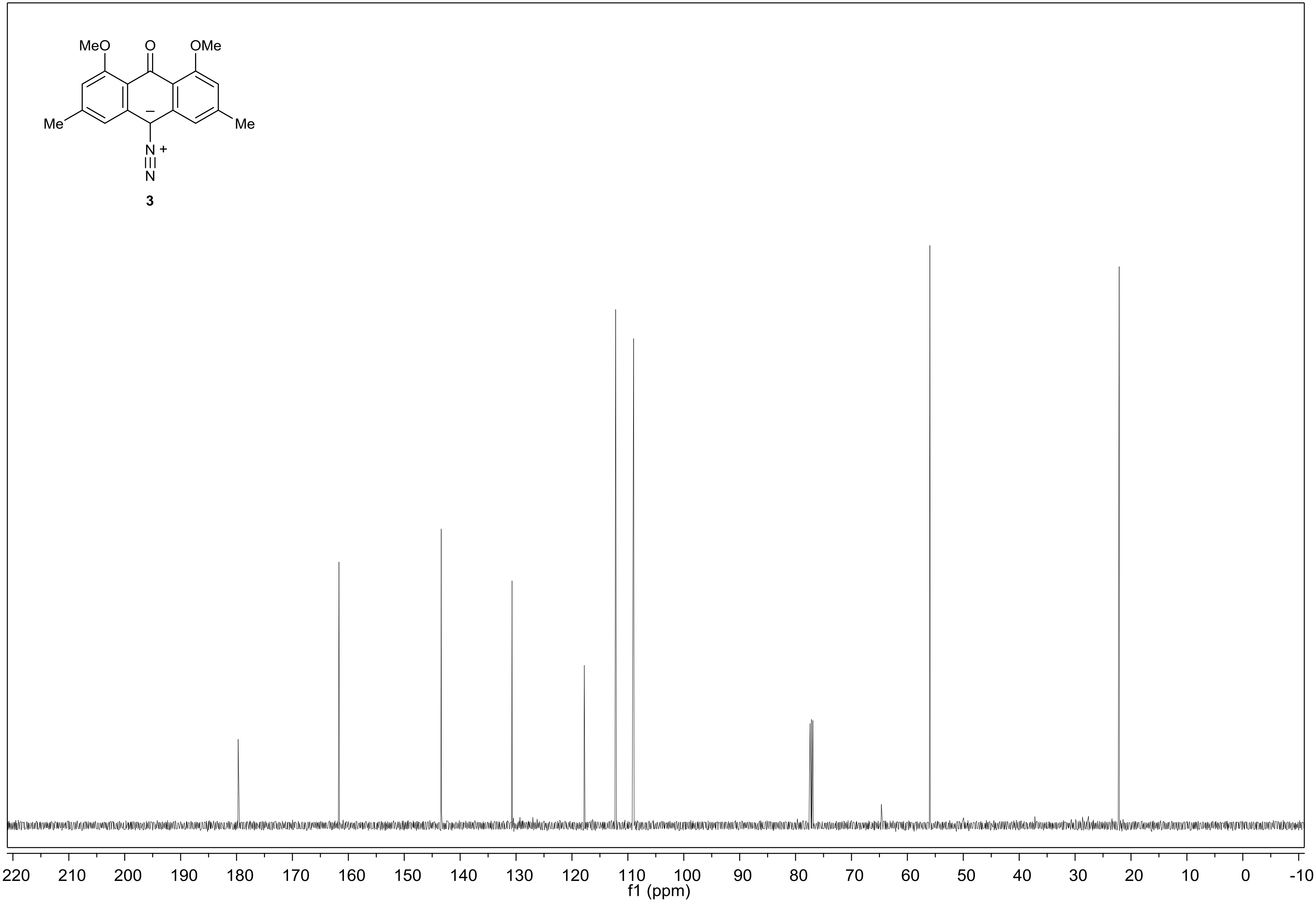

Supplementary Figure 5. ^{1}H NMR Spectrum of 13 (400 MHz, $CDCl_3$)

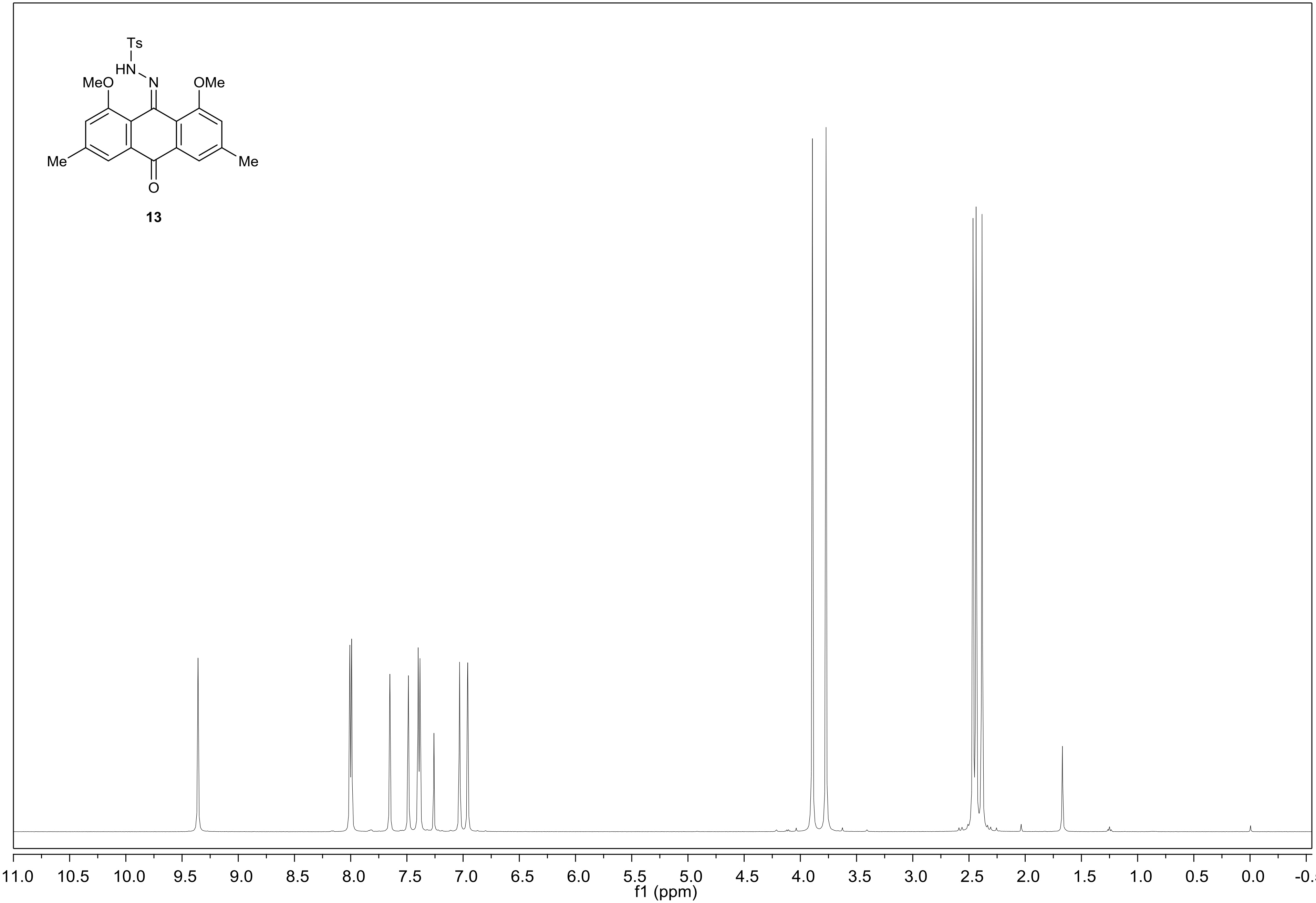

Supplementary Figure 6. ^{13}C NMR Spectrum of 13 (126 MHz, $CDCl_3$)

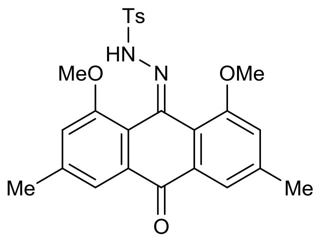

13

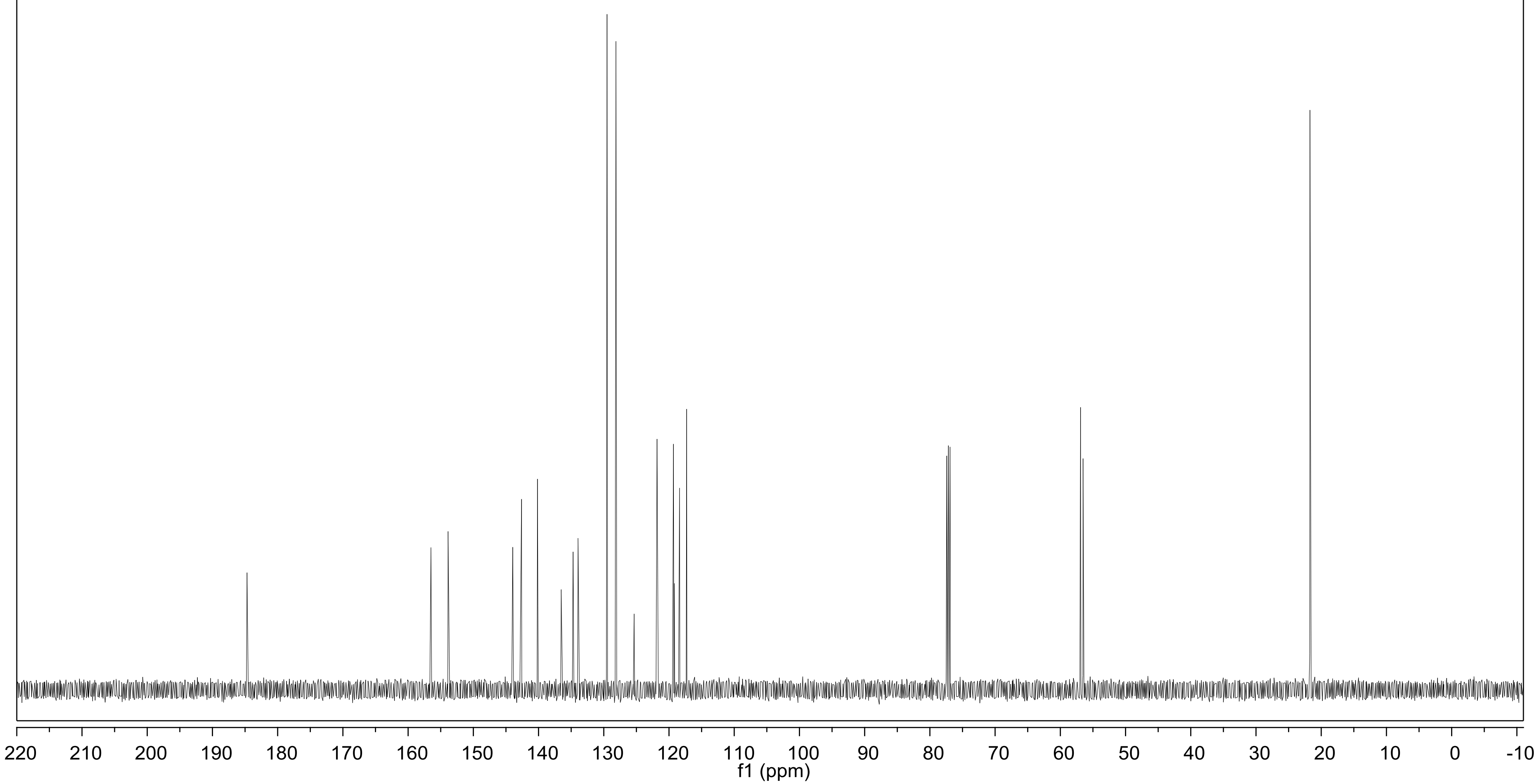

Supplementary Figure 7. ^{1}H NMR Spectrum of 14 (400 MHz, $CDCl_3$)

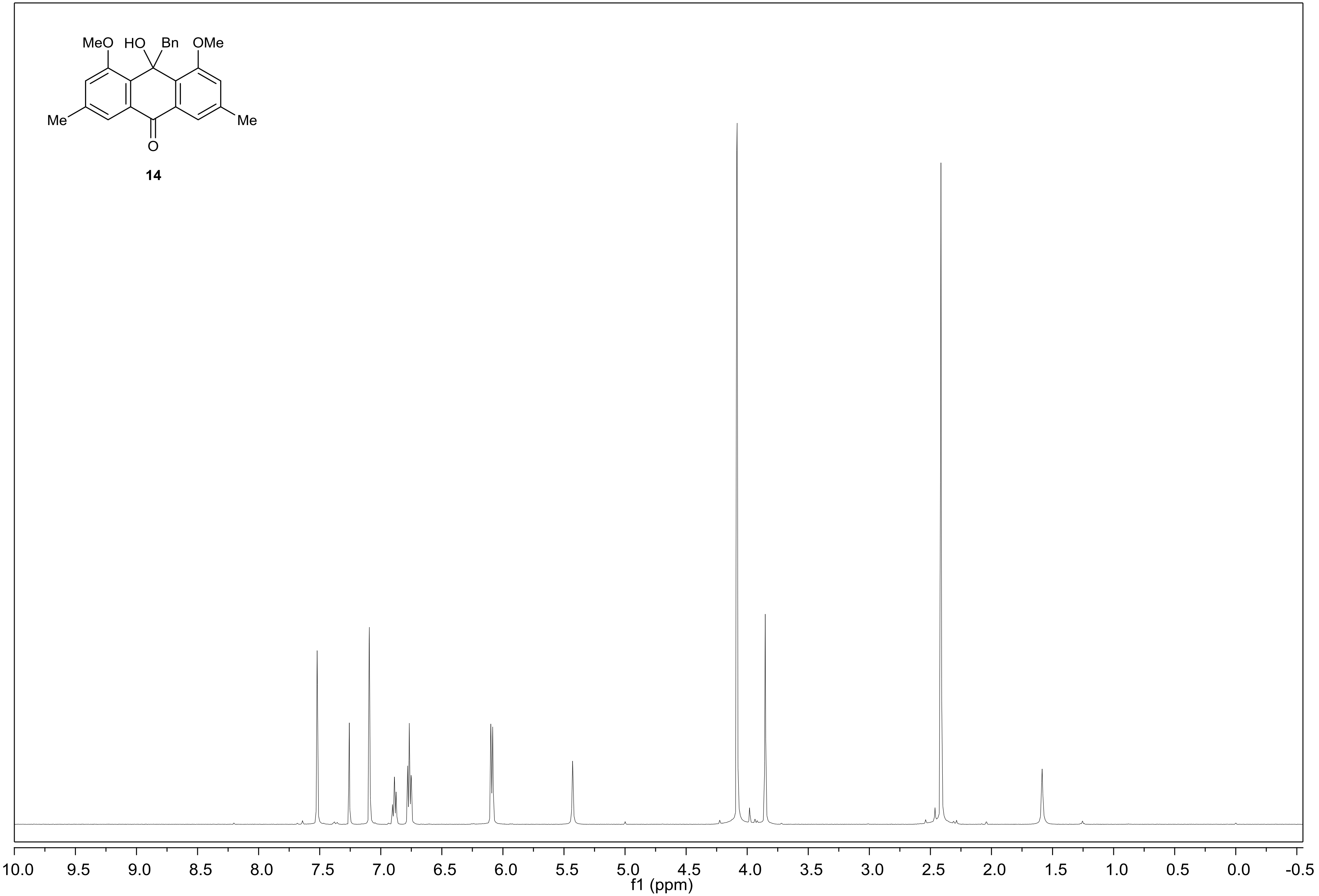

Supplementary Figure 8. ^{13}C NMR Spectrum of 14 (126 MHz, $CDCl_3$)

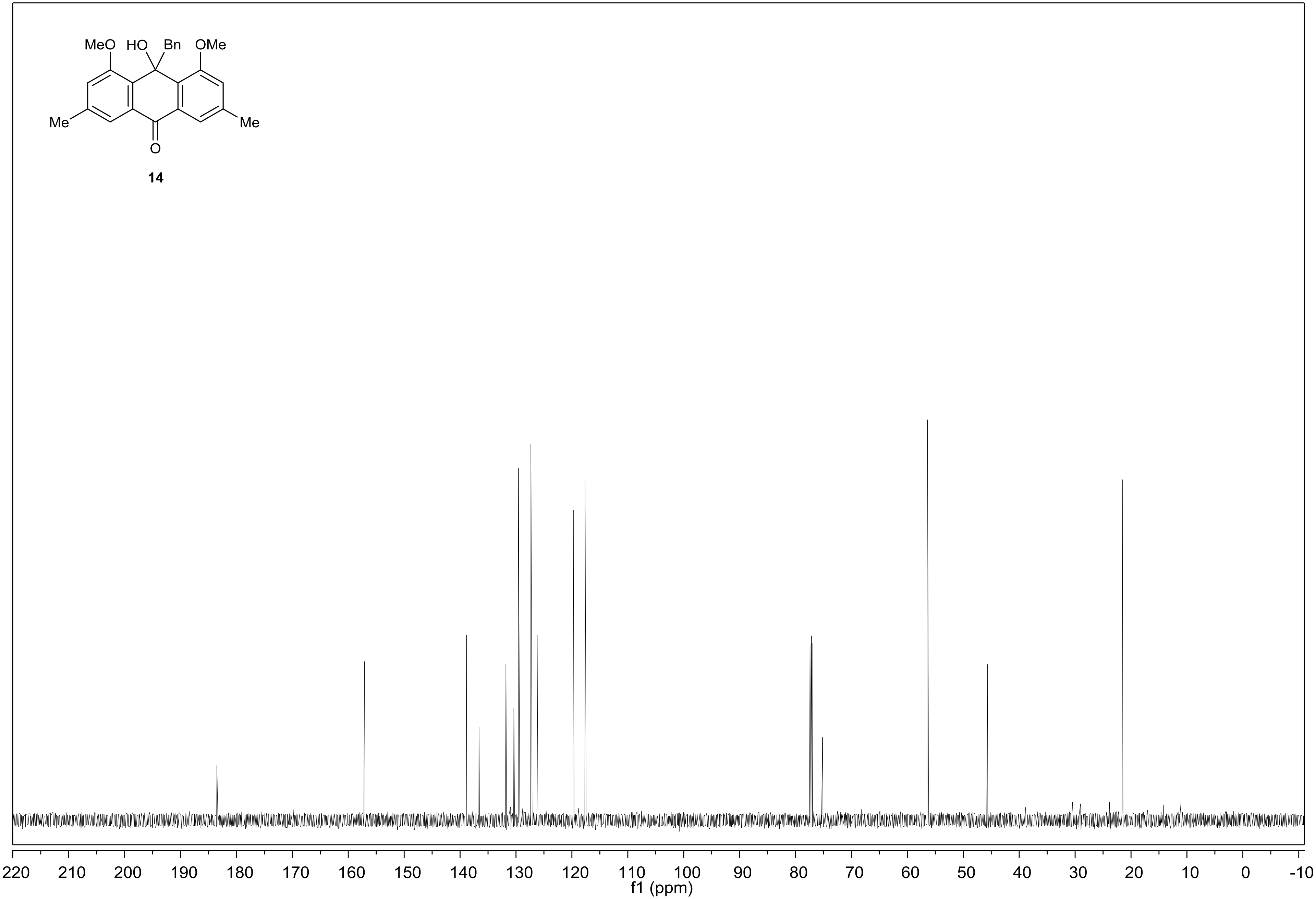

Supplementary Figure 9. ^{1}H NMR Spectrum of 15 (400 MHz, $CDCl_3$)

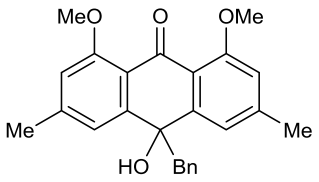

15

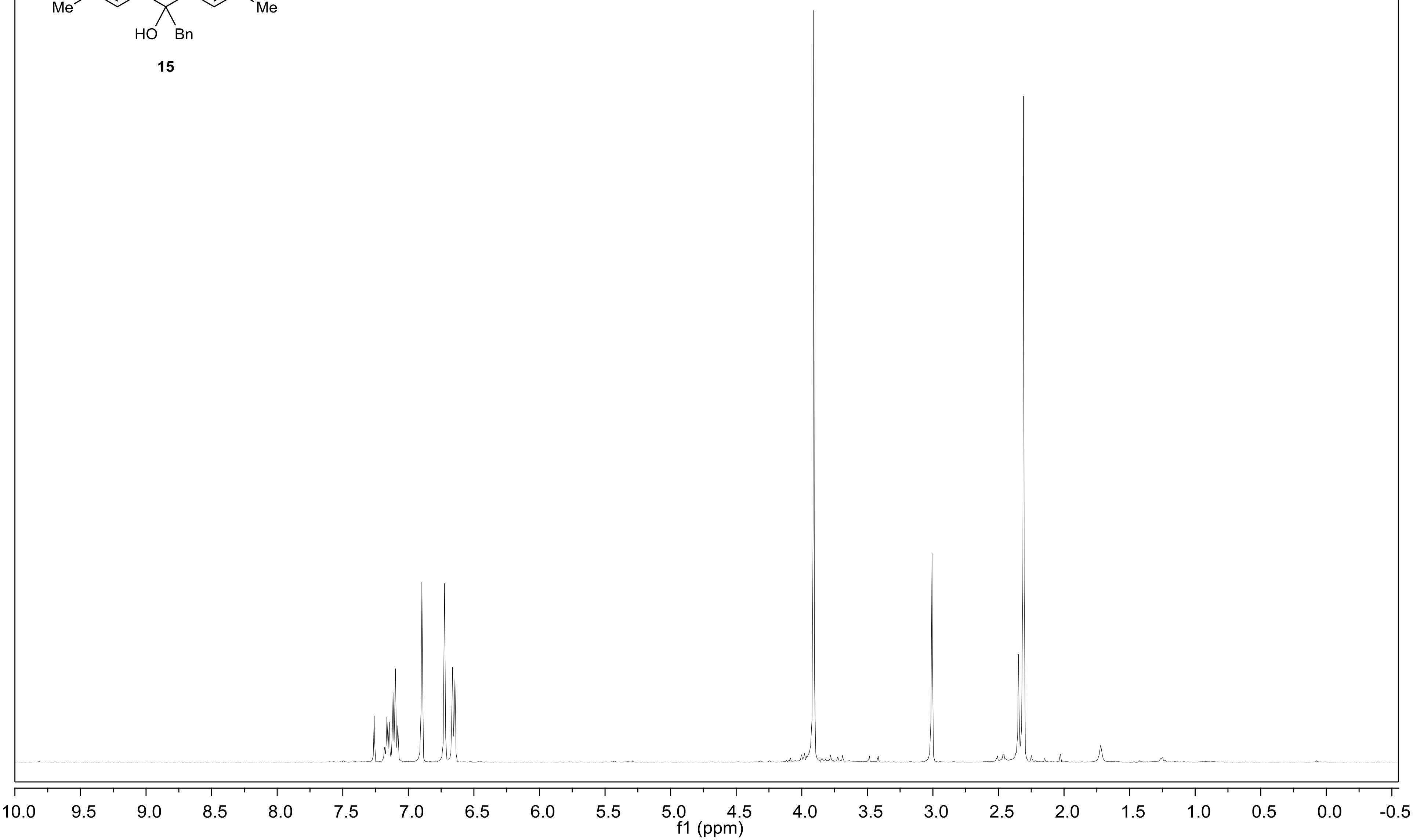

Supplementary Figure 10. ^{13}**C NMR Spectrum of 15 (101 MHz,** $CDCl_3$**)**

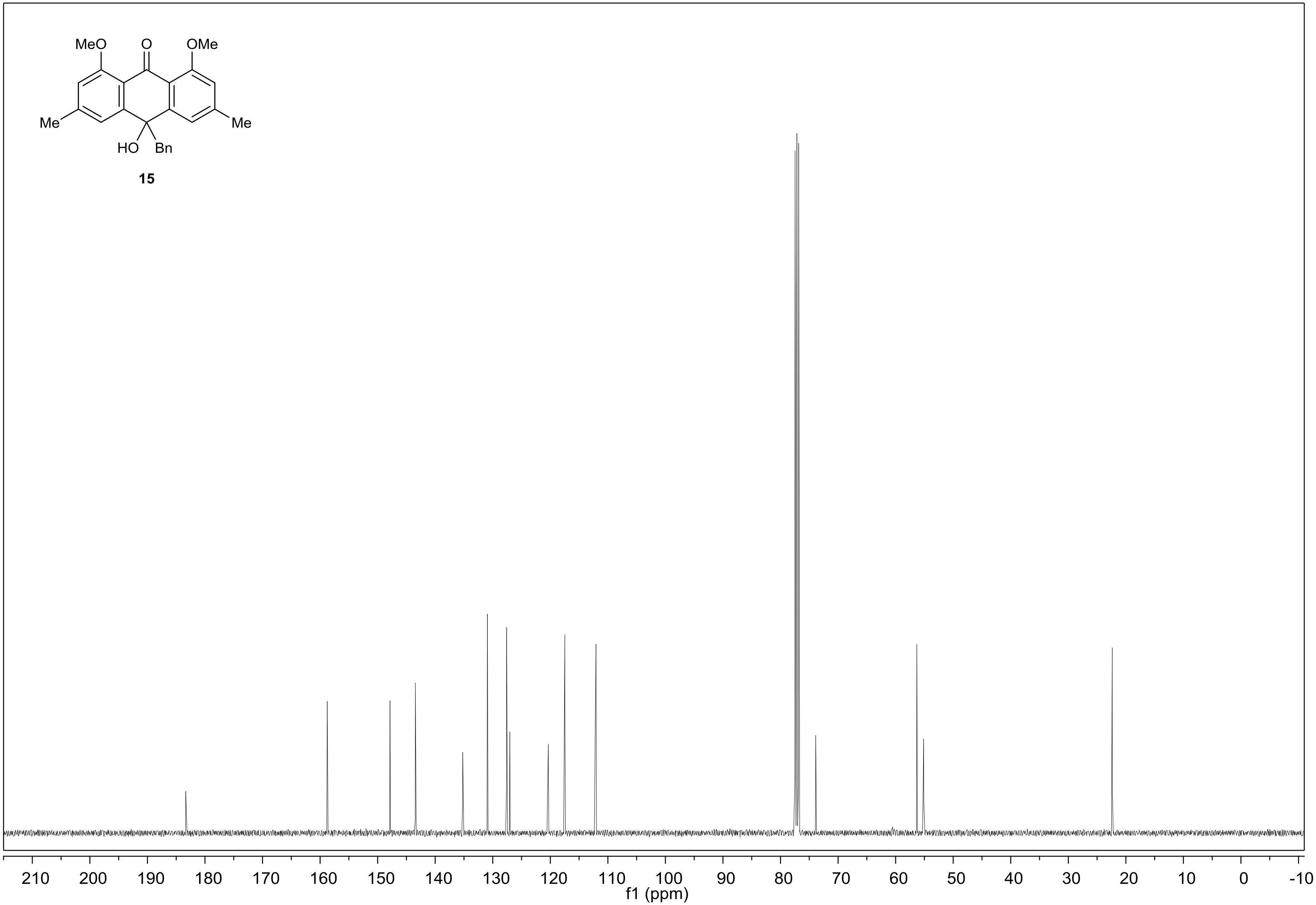

Supplementary Figure 11. ^{1}**H NMR Spectrum of 17 (500 MHz, CDCl$_3$)**

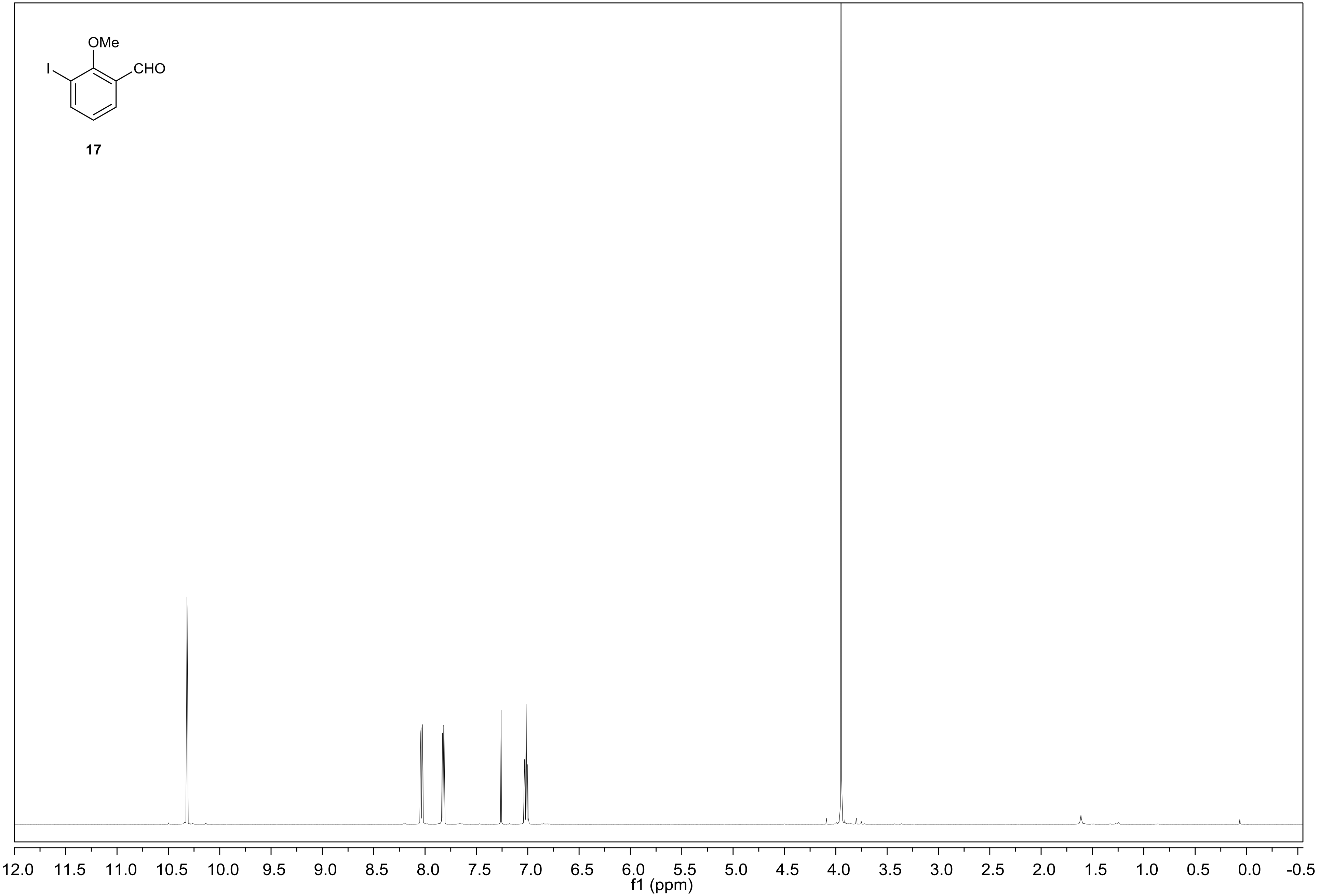

Supplementary Figure 12. ^{13}C NMR Spectrum of 17 (126 MHz, $CDCl_3$)

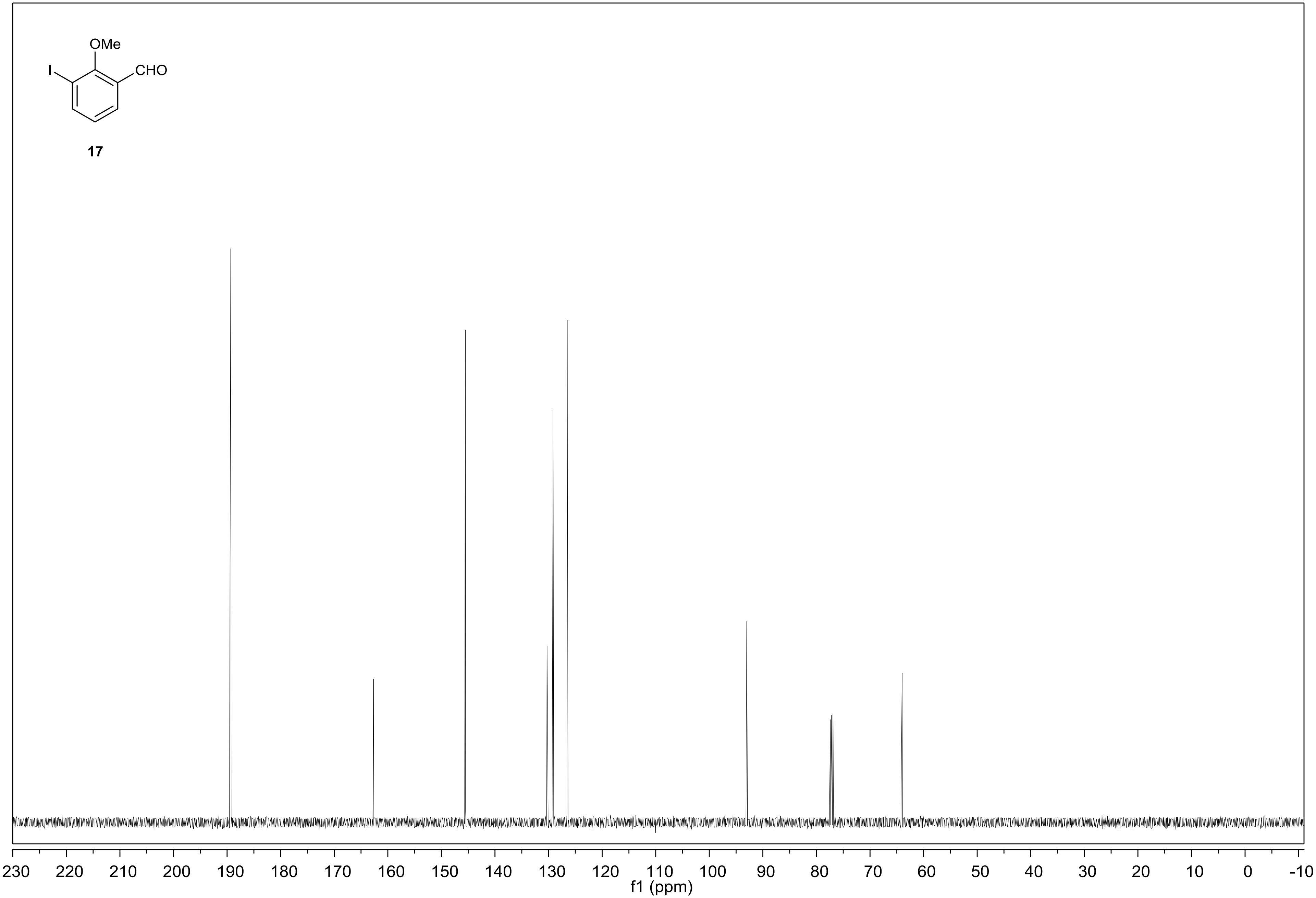

Supplementary Figure 13. ^{1}H NMR Spectrum of 18 (500 MHz, $CDCl_3$)

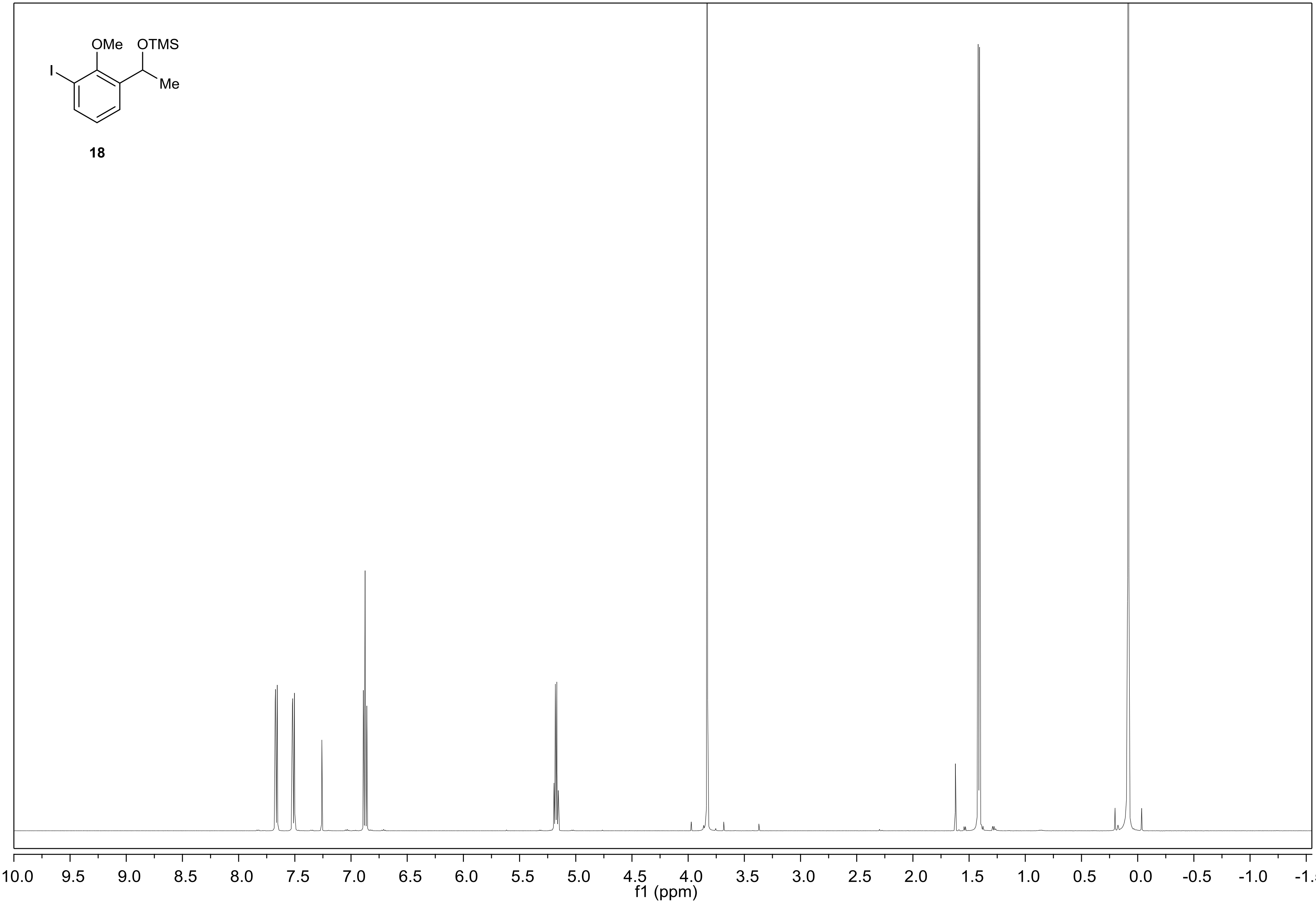

Supplementary Figure 14. ^{13}C NMR Spectrum of 18 (126 MHz, $CDCl_3$)

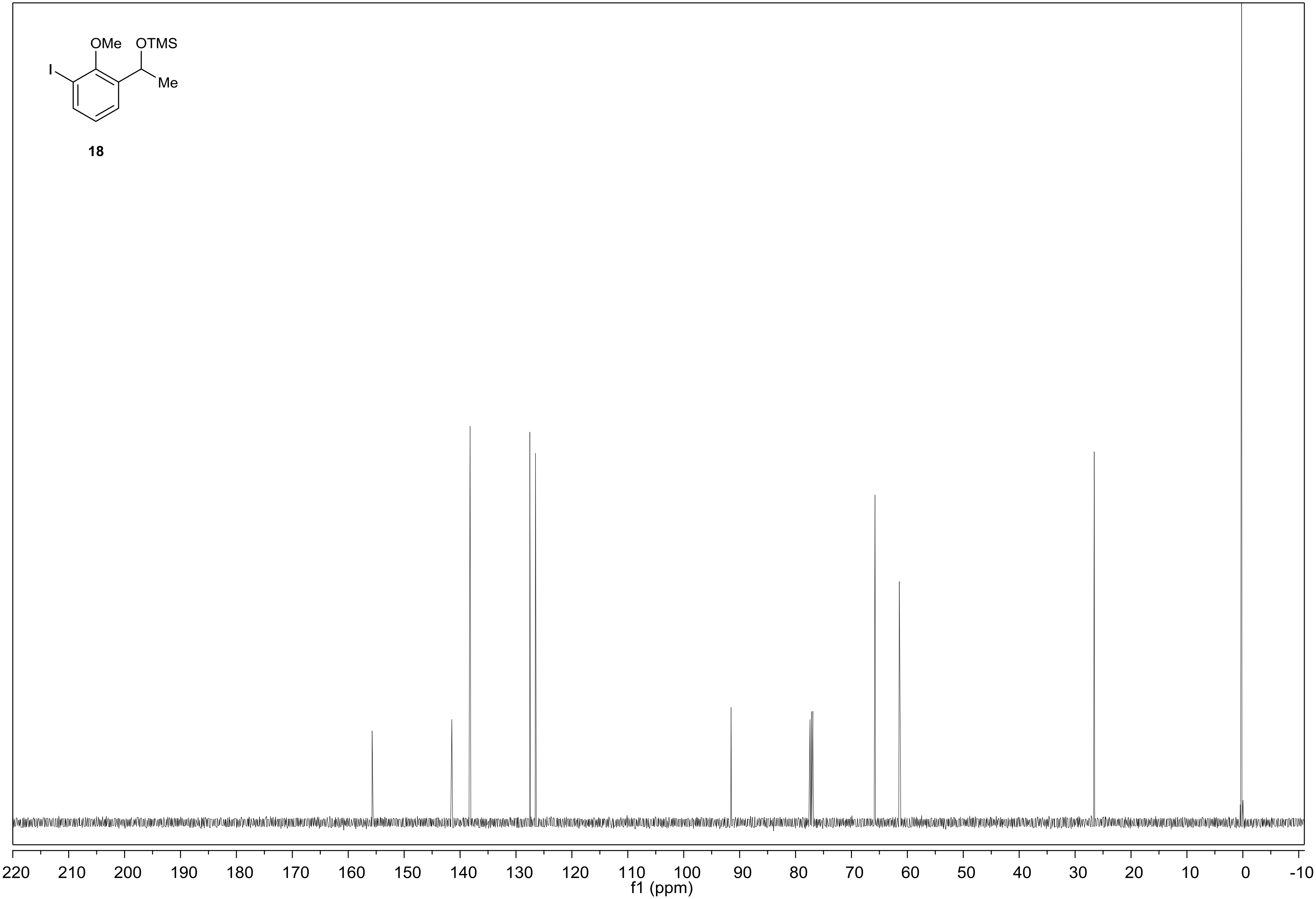

Supplementary Figure 15. ^{1}H NMR Spectrum of 20 (400 MHz, $CDCl_3$)

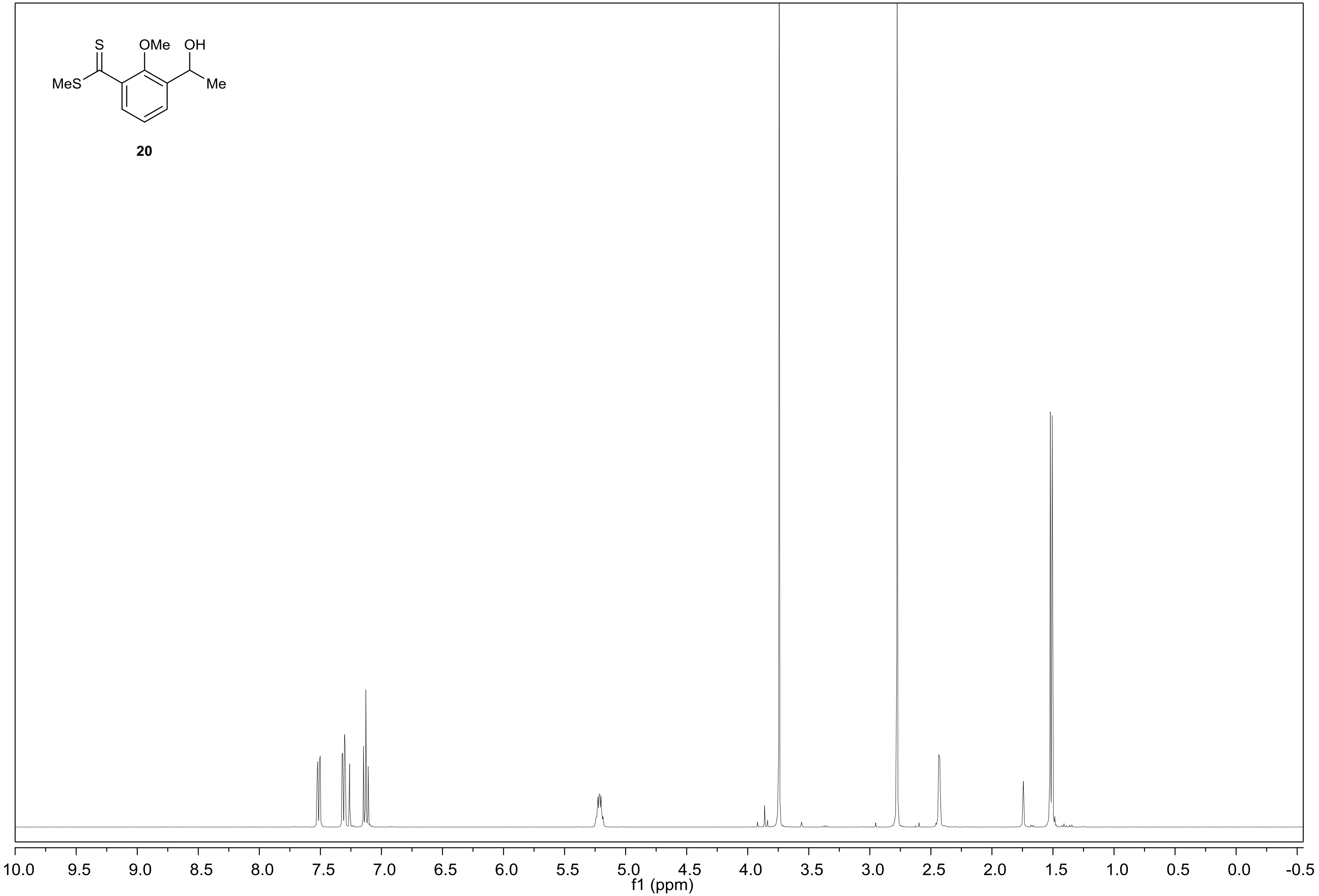

Supplementary Figure 16. ^{13}C NMR Spectrum of 20 (126 MHz, $CDCl_3$)

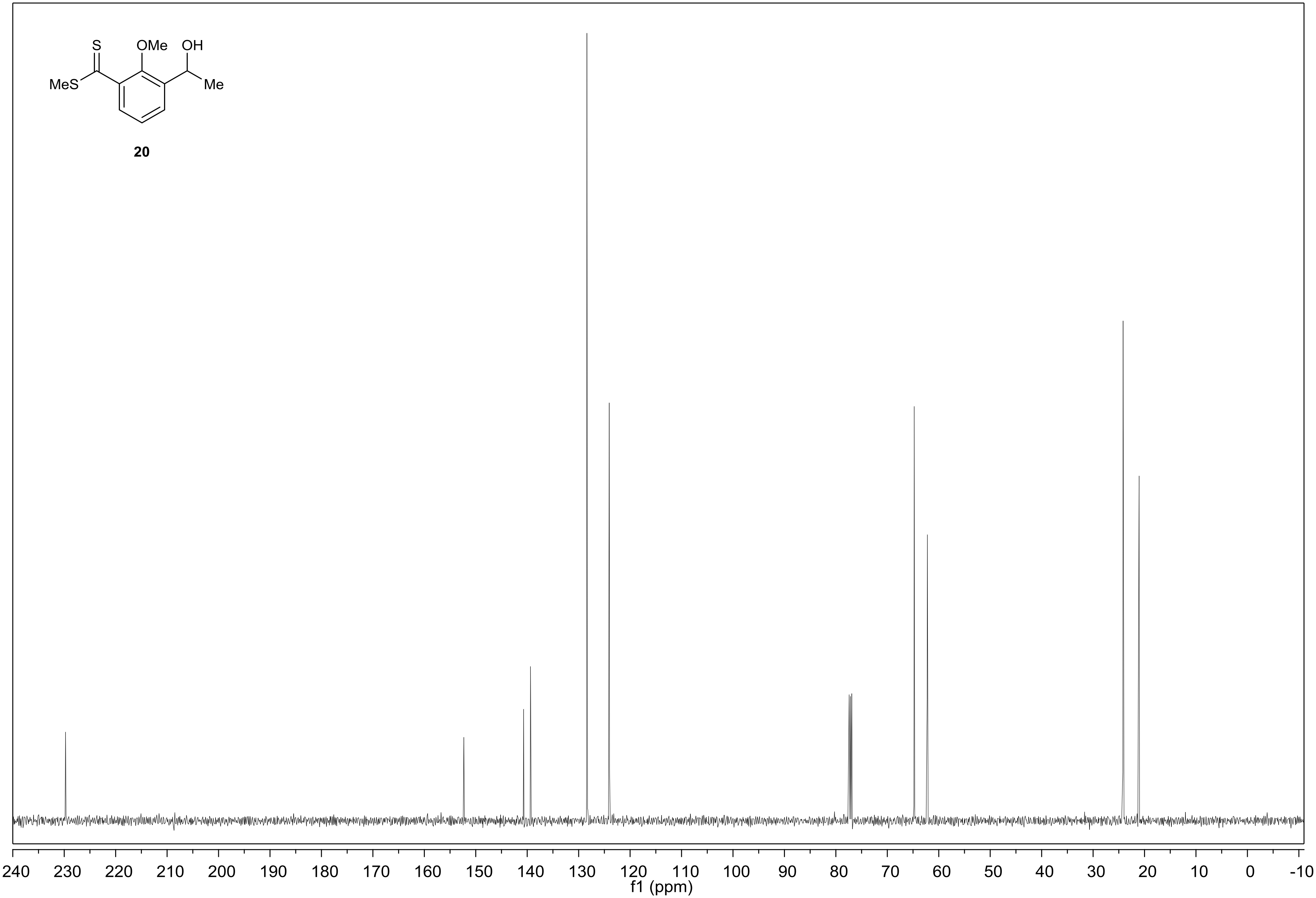

Supplementary Figure 17. ^{1}H NMR Spectrum of 21 (400 MHz, $CDCl_3$)

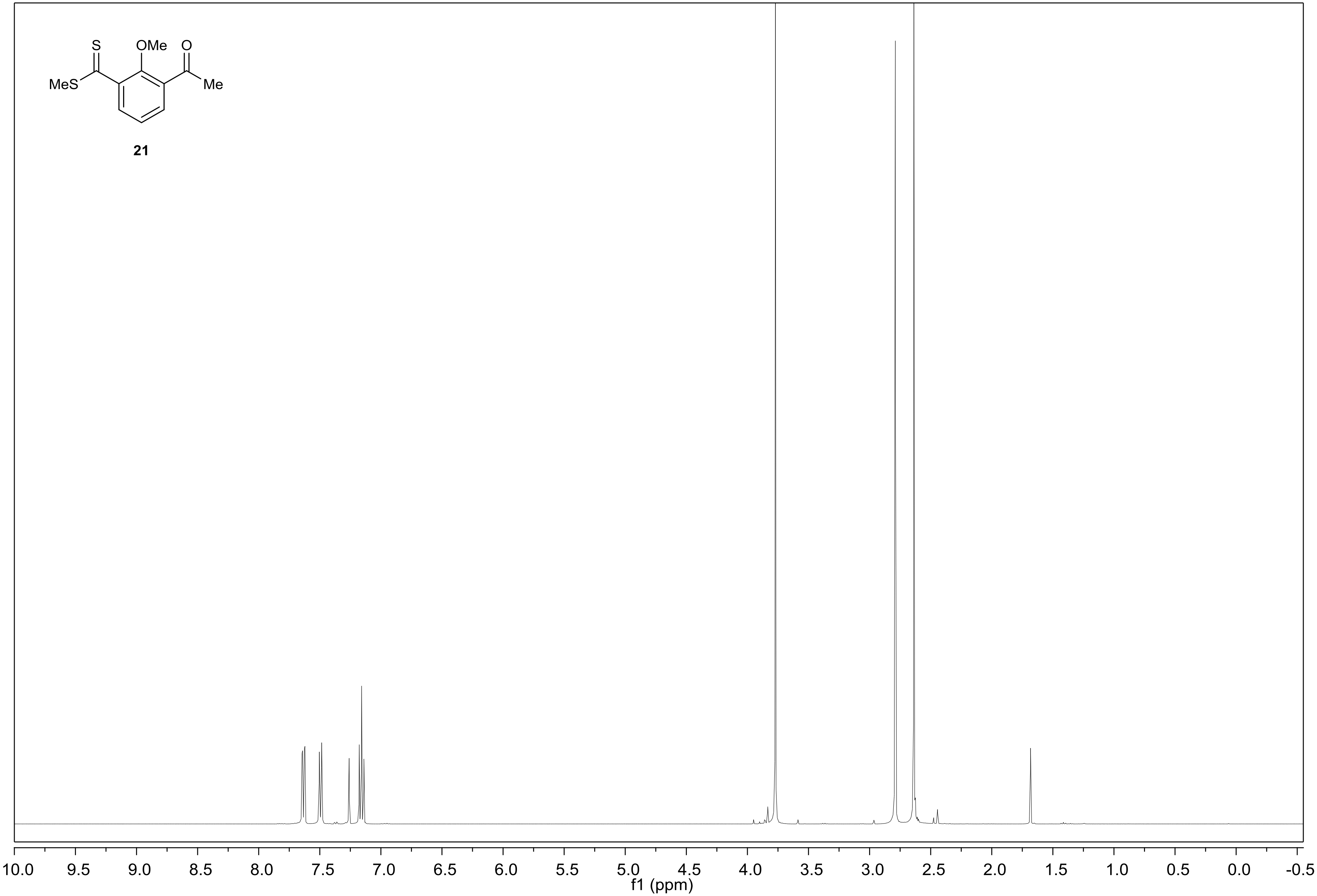

Supplementary Figure 18. ^{13}C NMR Spectrum of 21 (126 MHz, $CDCl_3$)

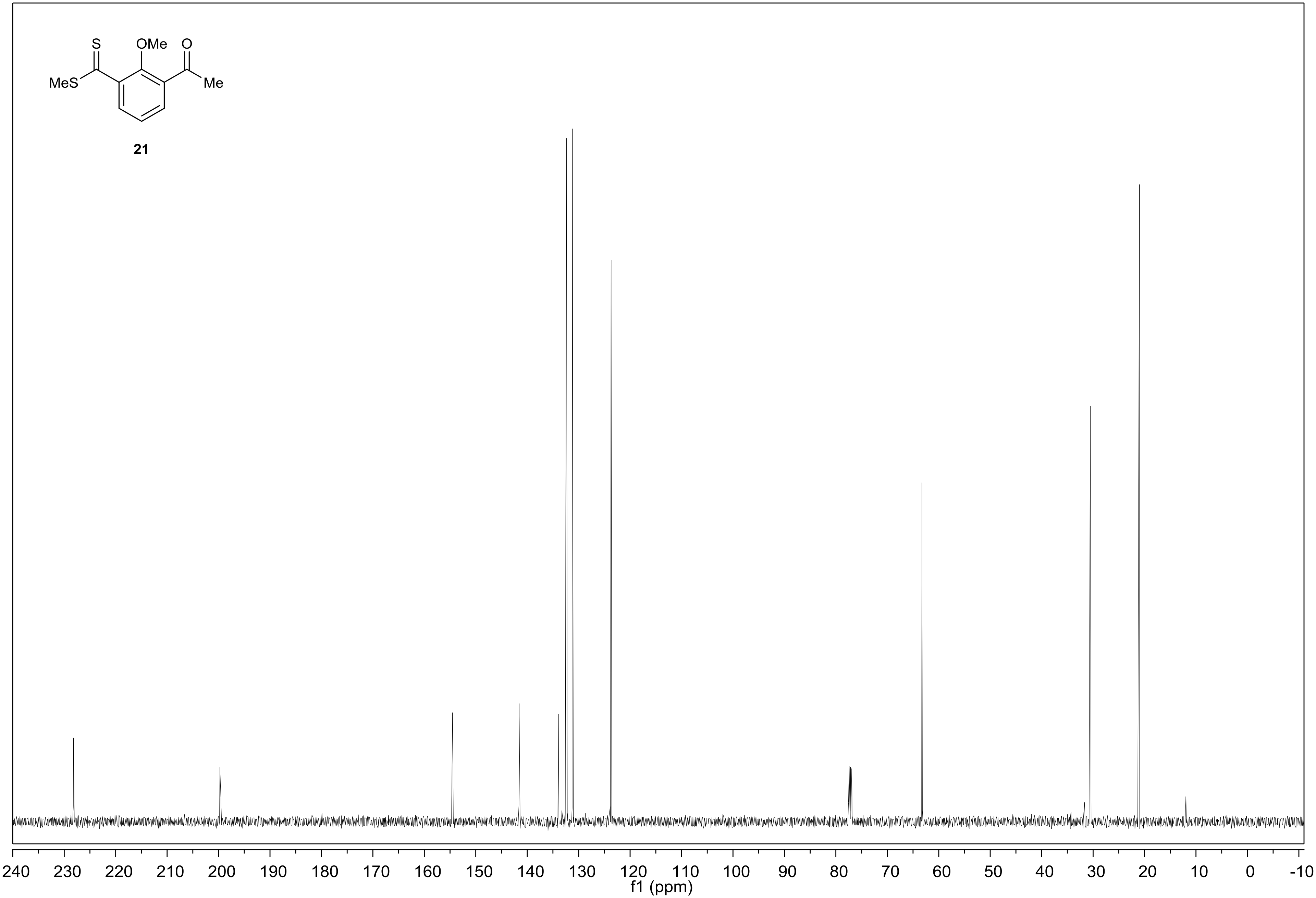

Supplementary Figure 19. ^{1}H NMR Spectrum of 4 (500 MHz, $CDCl_3$)

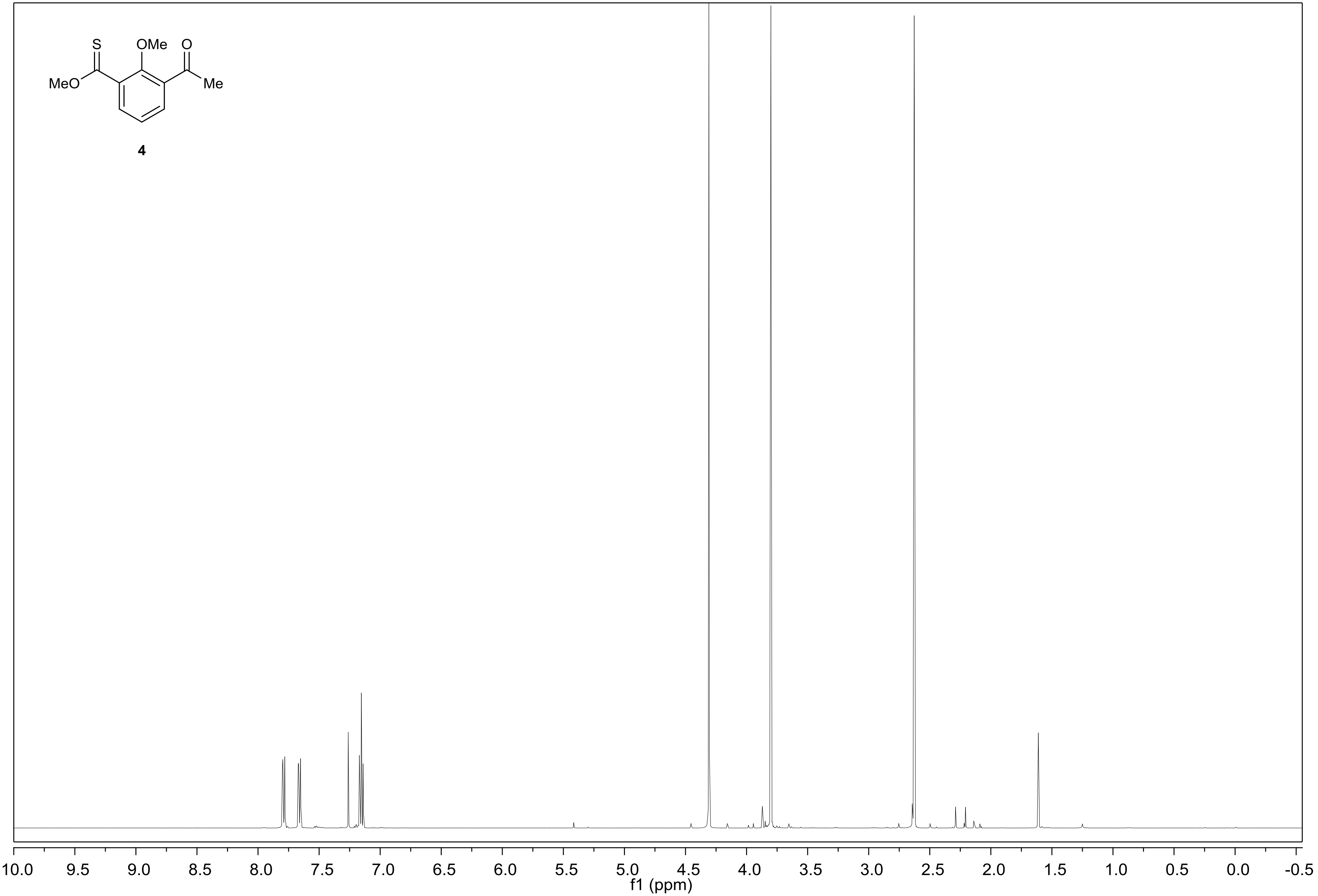

Supplementary Figure 20. ^{13}C NMR Spectrum of 4 (101 MHz, $CDCl_3$)

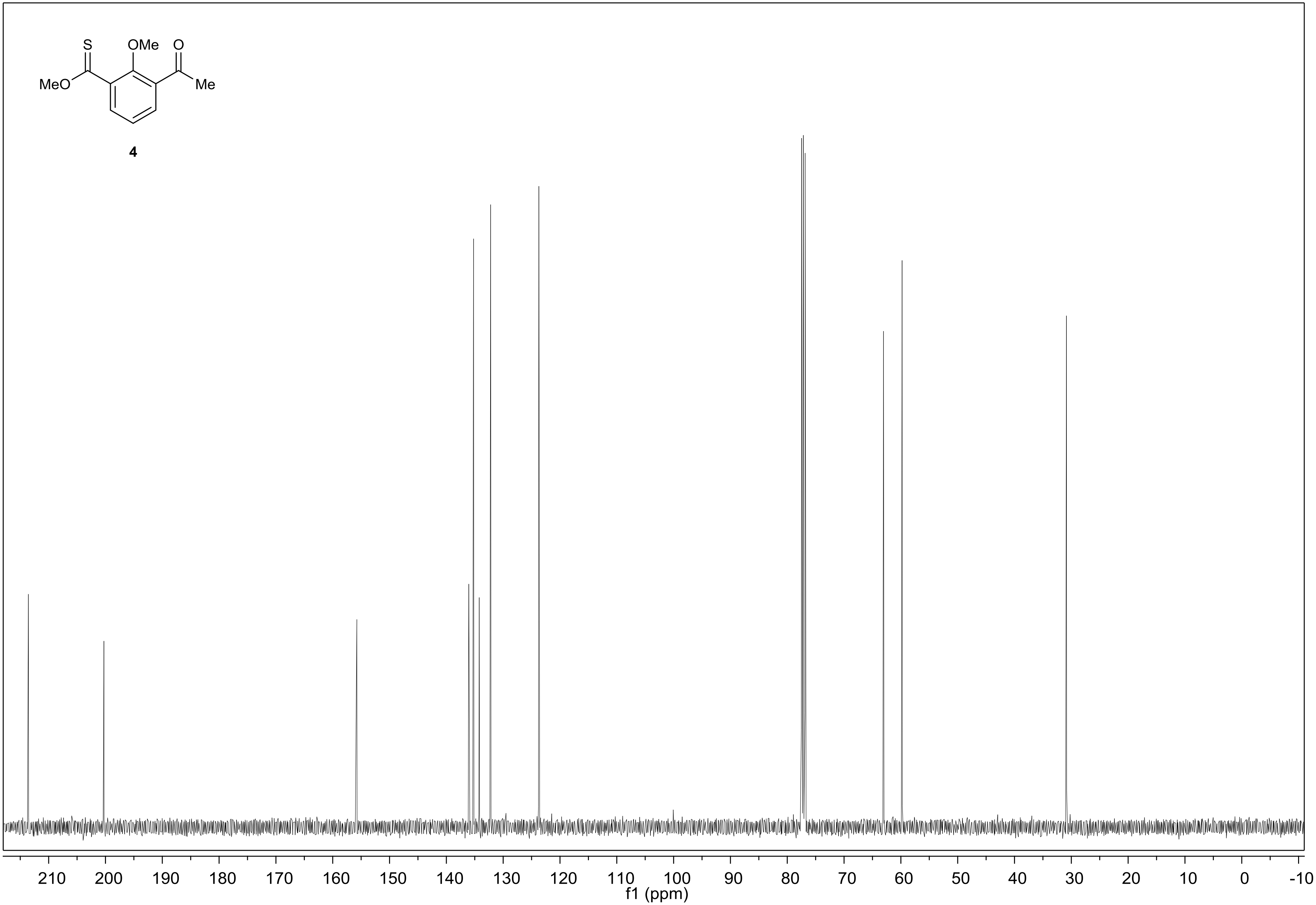

Supplementary Figure 21. ^{1}H NMR Spectrum of 2 (500 MHz, $CDCl_3$)

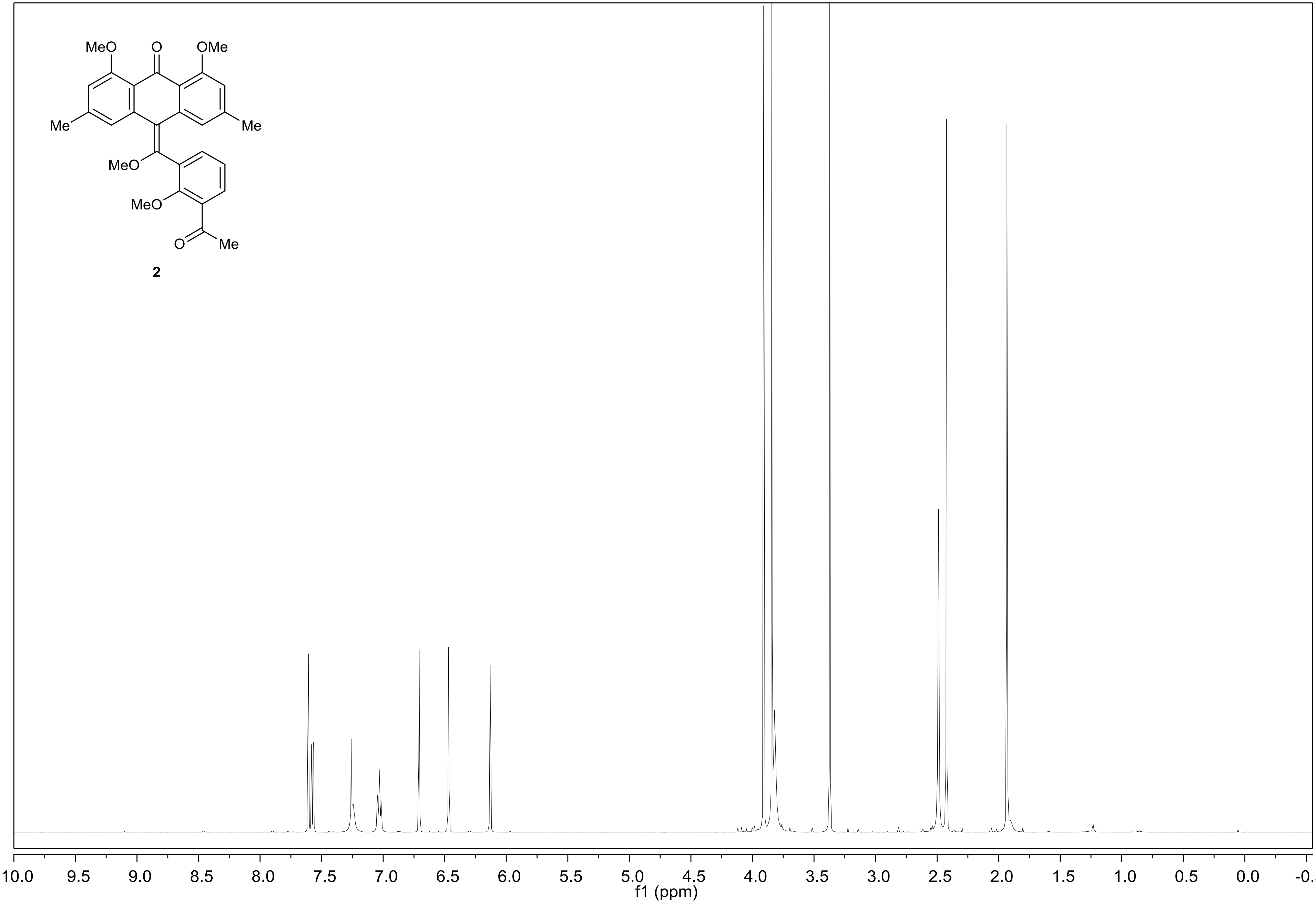

Supplementary Figure 22. ^{13}**C NMR Spectrum of 2 (126 MHz,** $CDCl_3$**)**

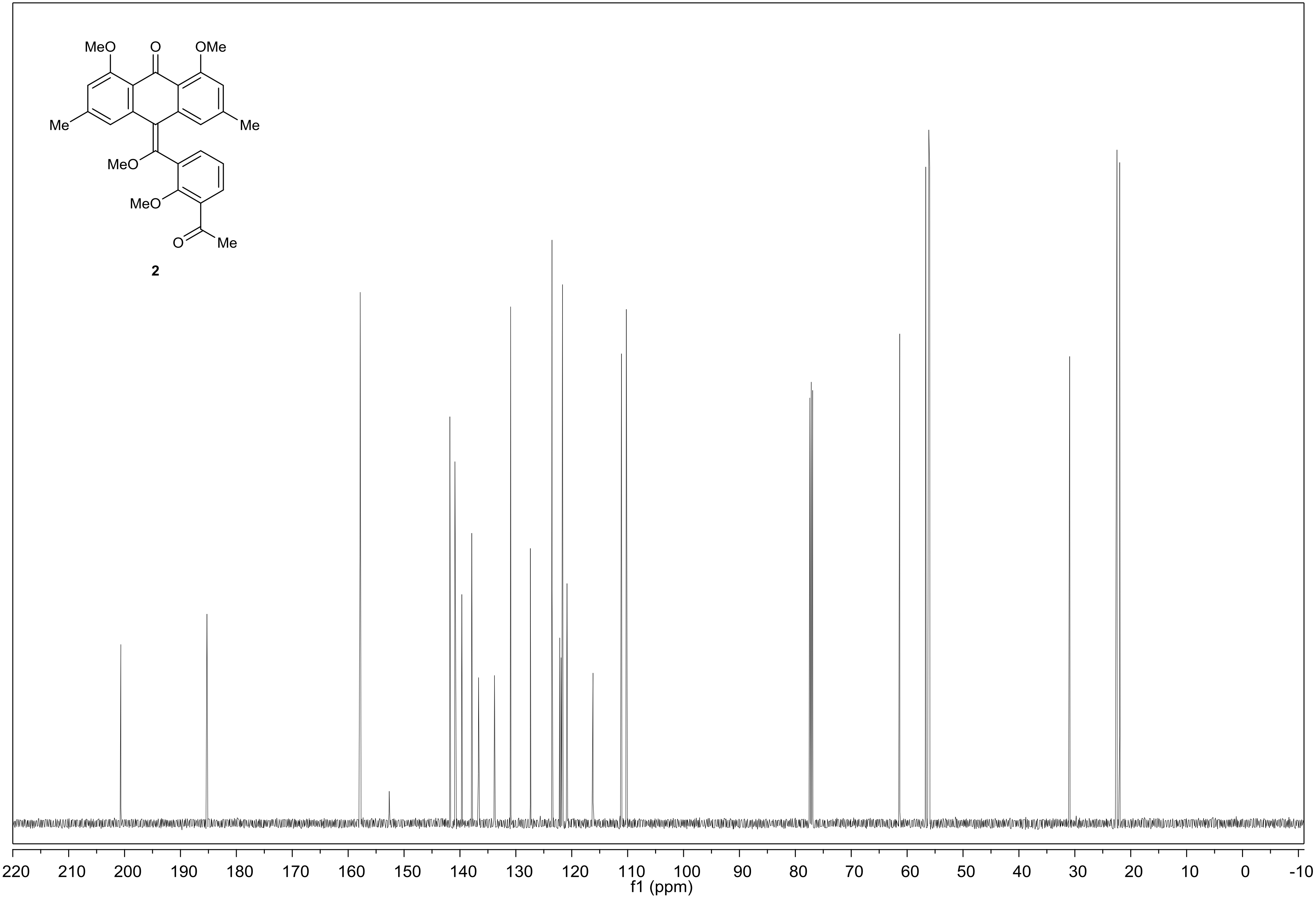

Supplementary Figure 23. ^{1}H NMR Spectrum of 24 (500 MHz, $CDCl_3$)

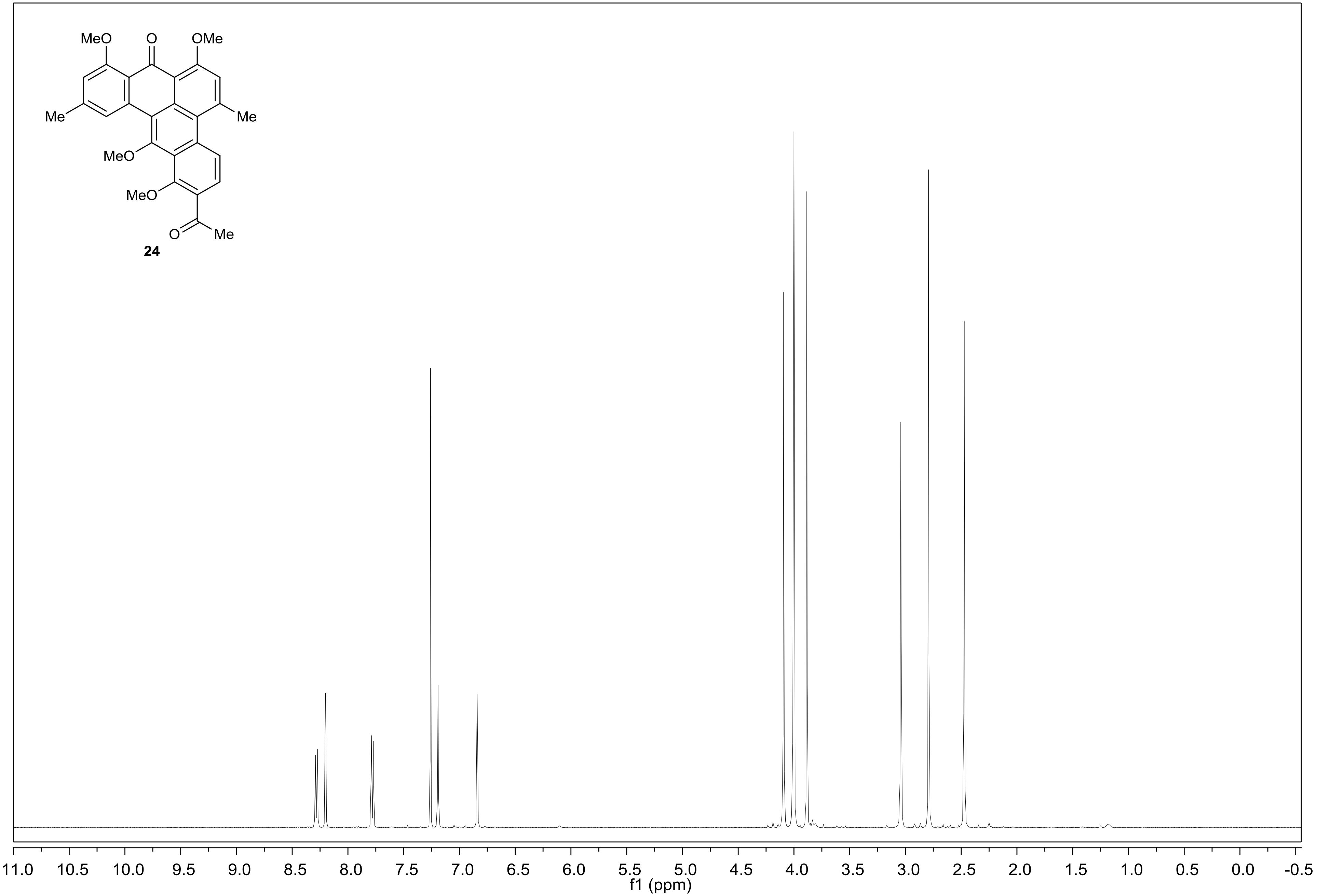

Supplementary Figure 24. ^{13}C NMR Spectrum of 24 (101 MHz, $CDCl_3$)

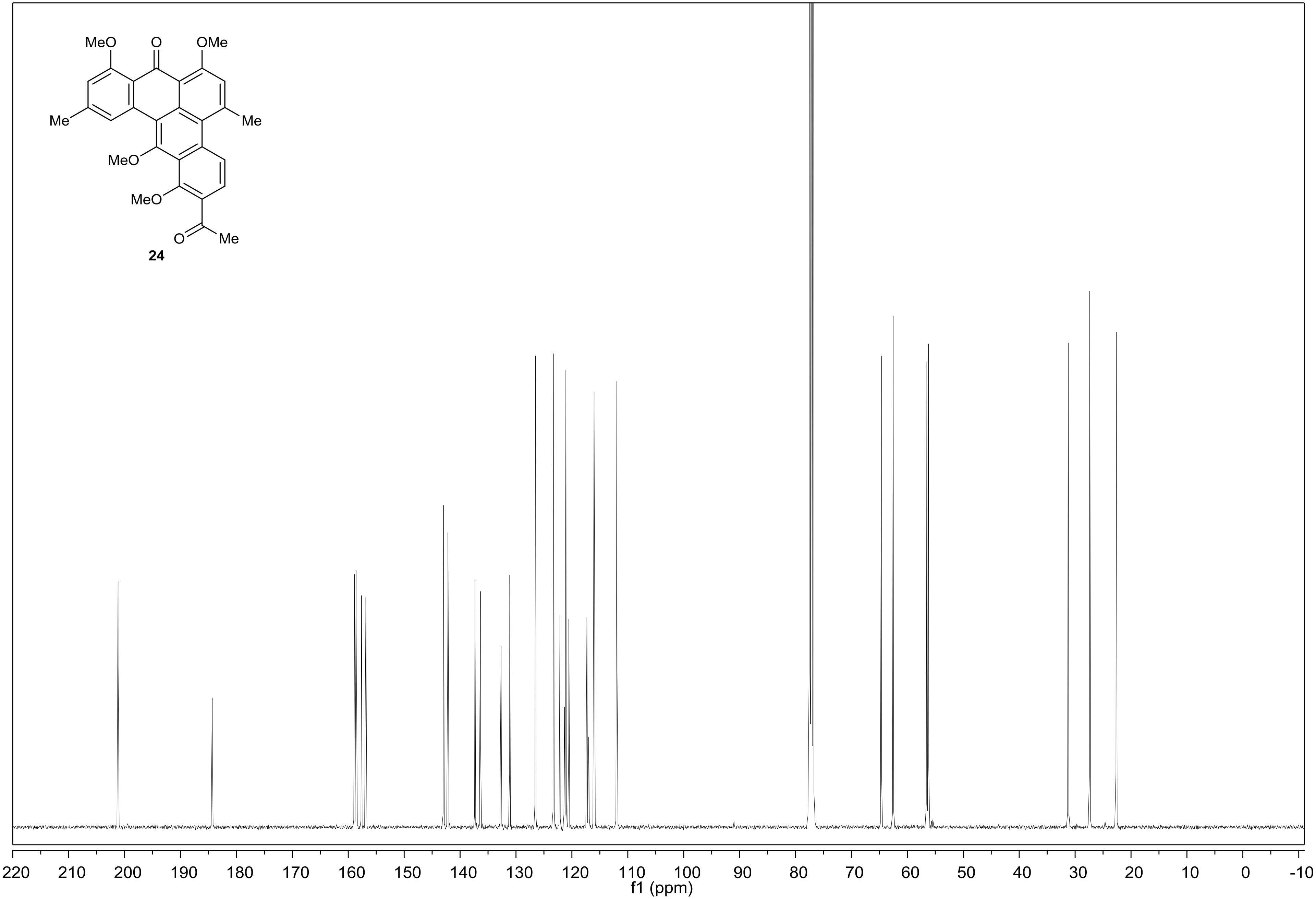

Supplementary Figure 25. ^{1}H NMR Spectrum of 1 (500 MHz, DMSO-d_6)

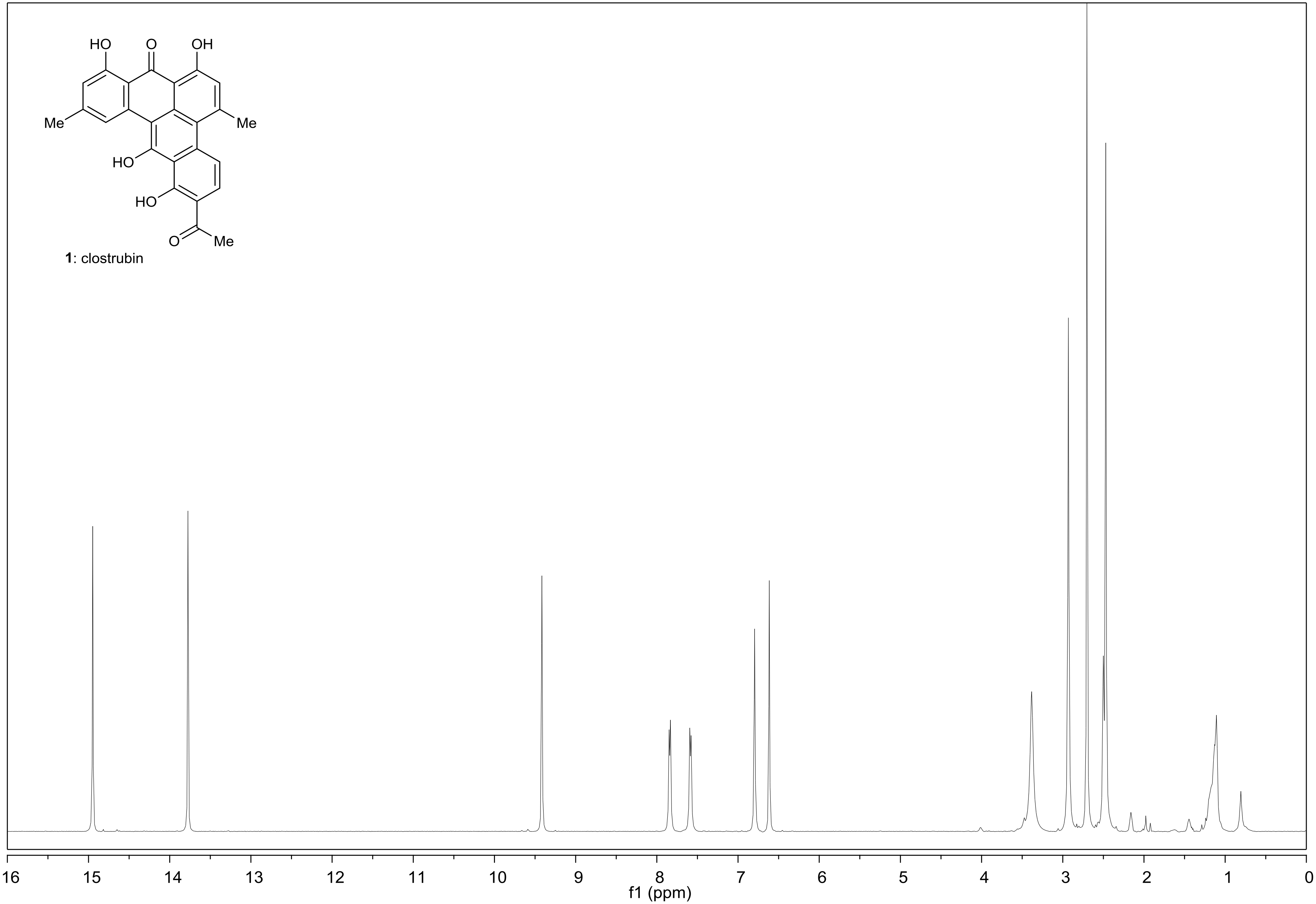

Supplementary Figure 26. ^{13}C NMR Spectrum of 1 (126 MHz, DMSO-d$_6$)

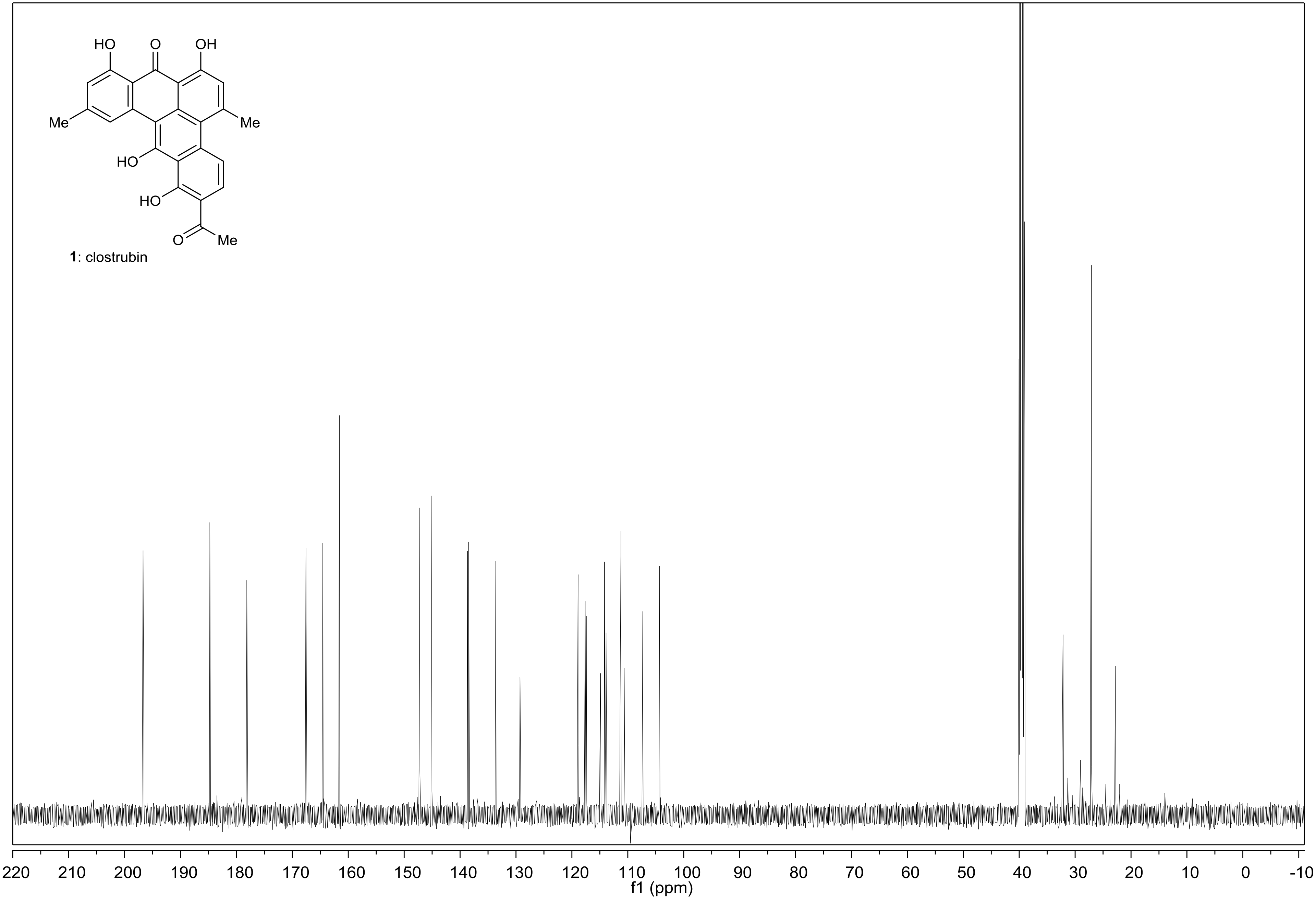

Supplementary Figure 27. HMQC spectrum of 1

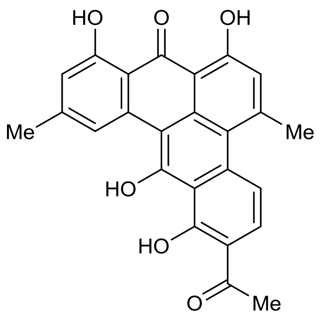

1: clostrubin

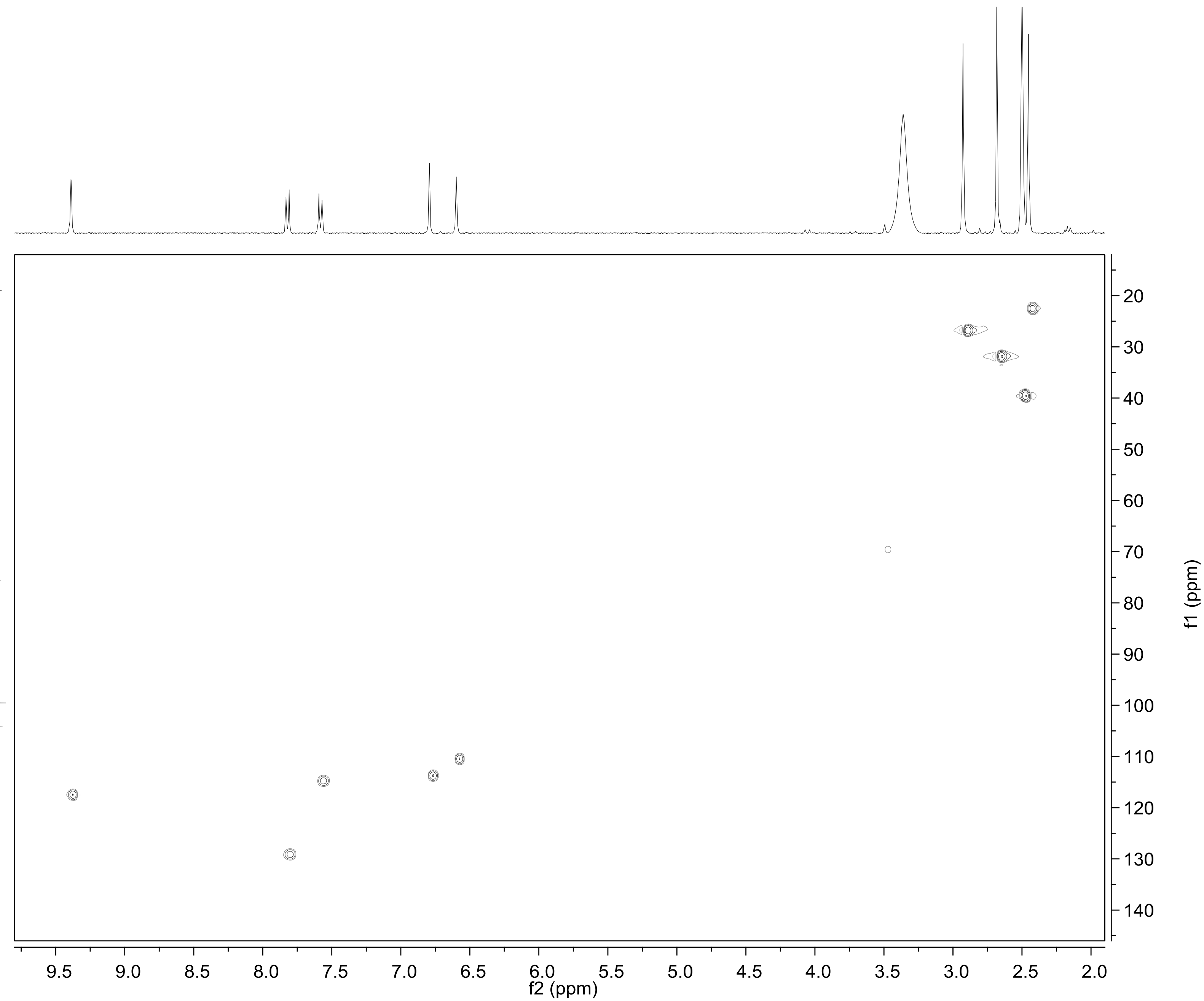

Supplementary Figure 28. HMBC spectrum of 1

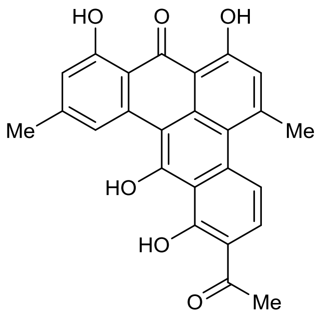

1: clostrubin

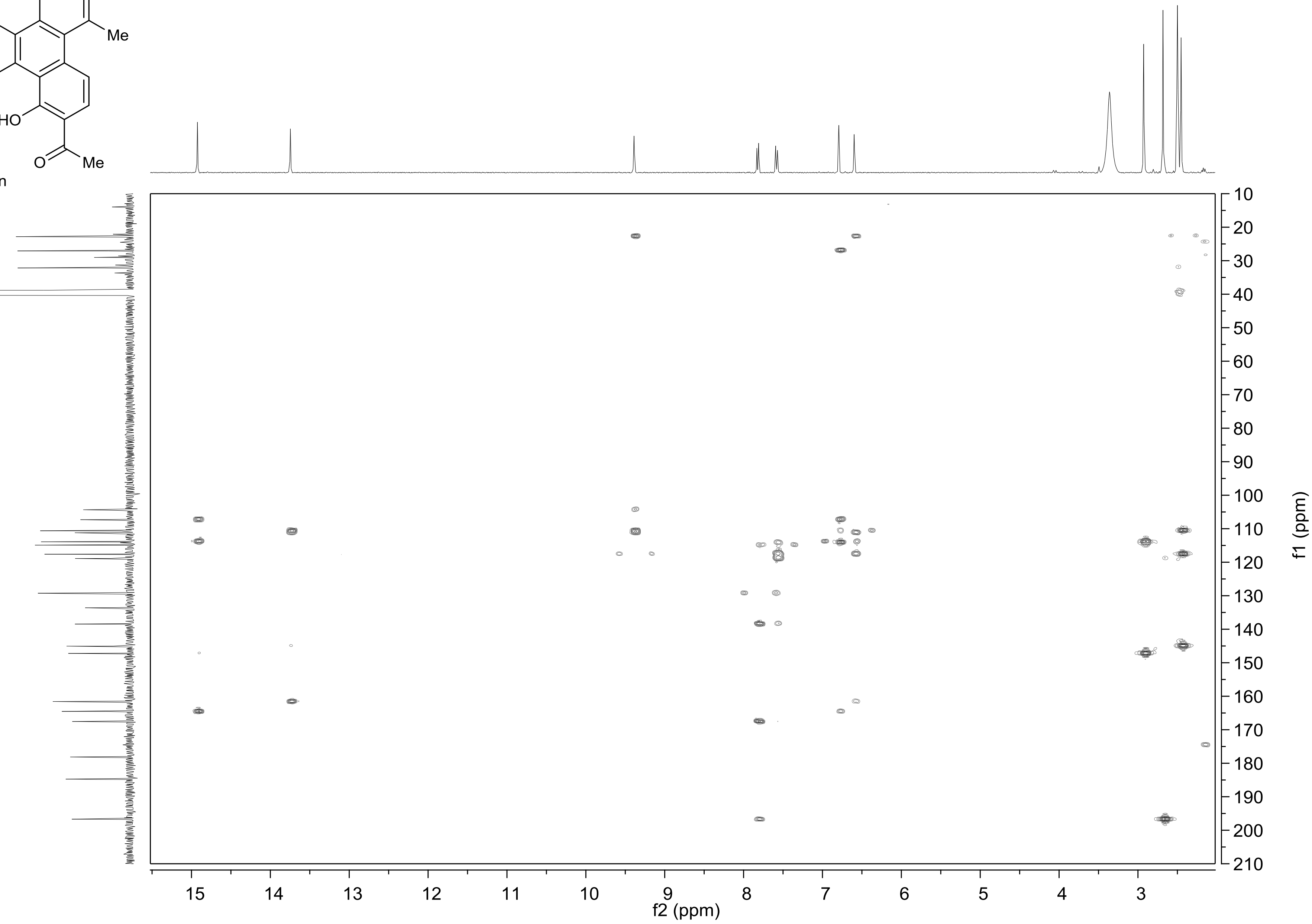

Supplementary Figure 29. NOESY Spectrum of 14

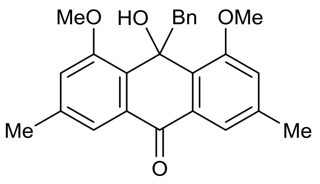

14

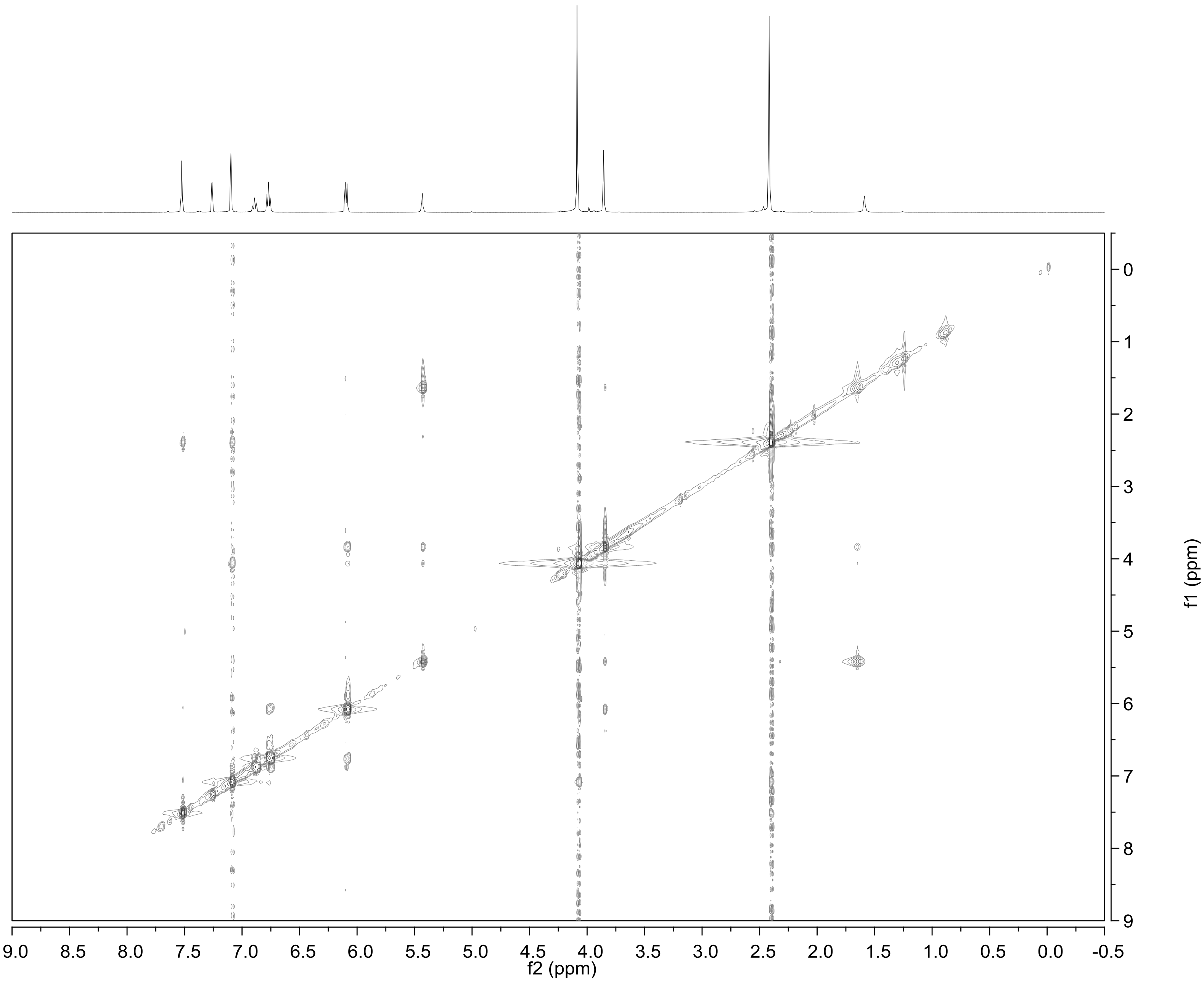

Supplementary Figure 30. NOESY Spectrum of 15

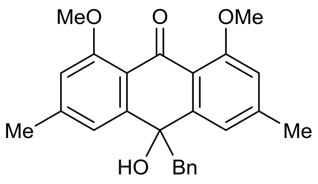

15

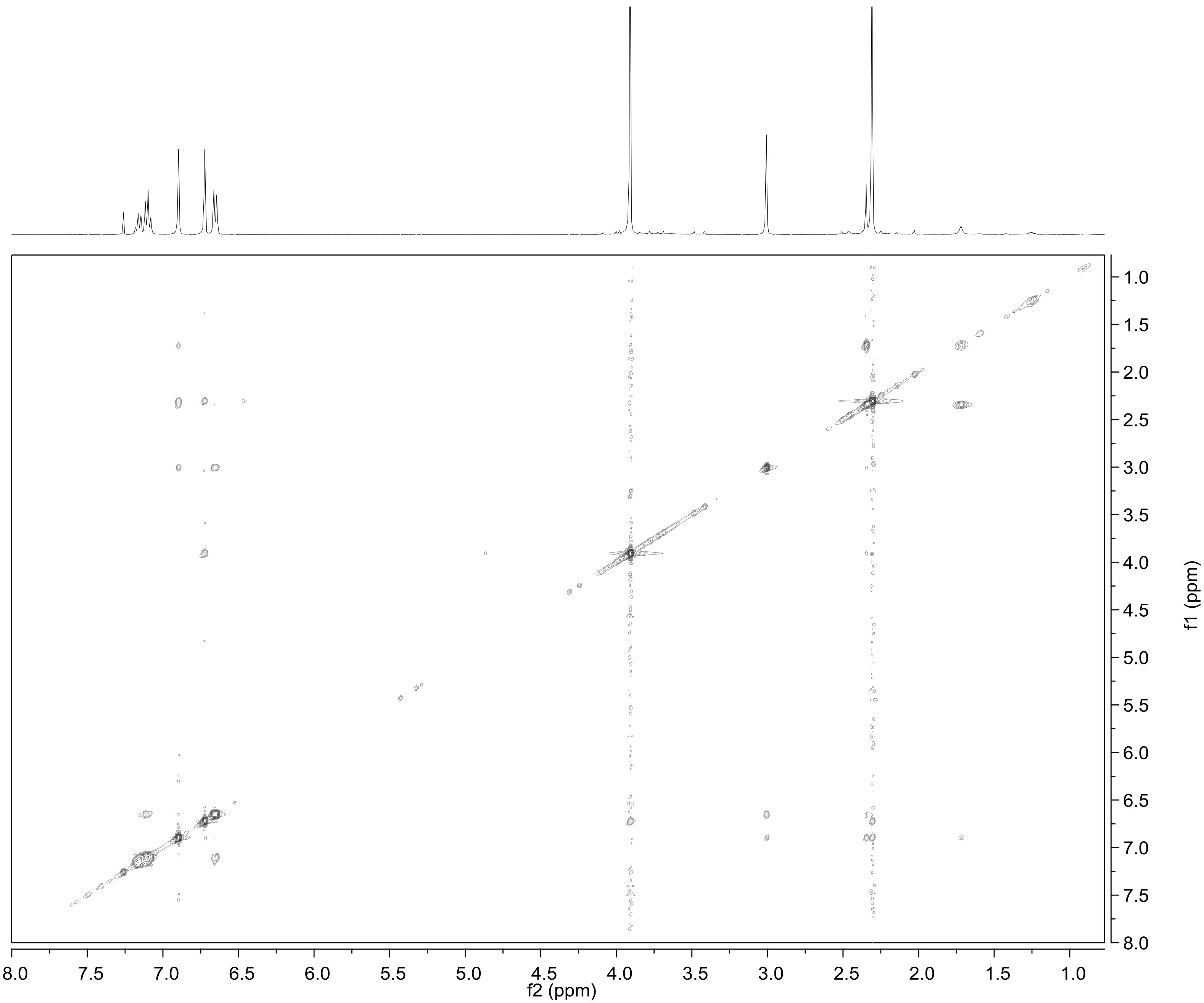

Supplementary Figure 31. Comparison of the ^{1}H NMR Spectra of Natural and Synthetic Clostrubin

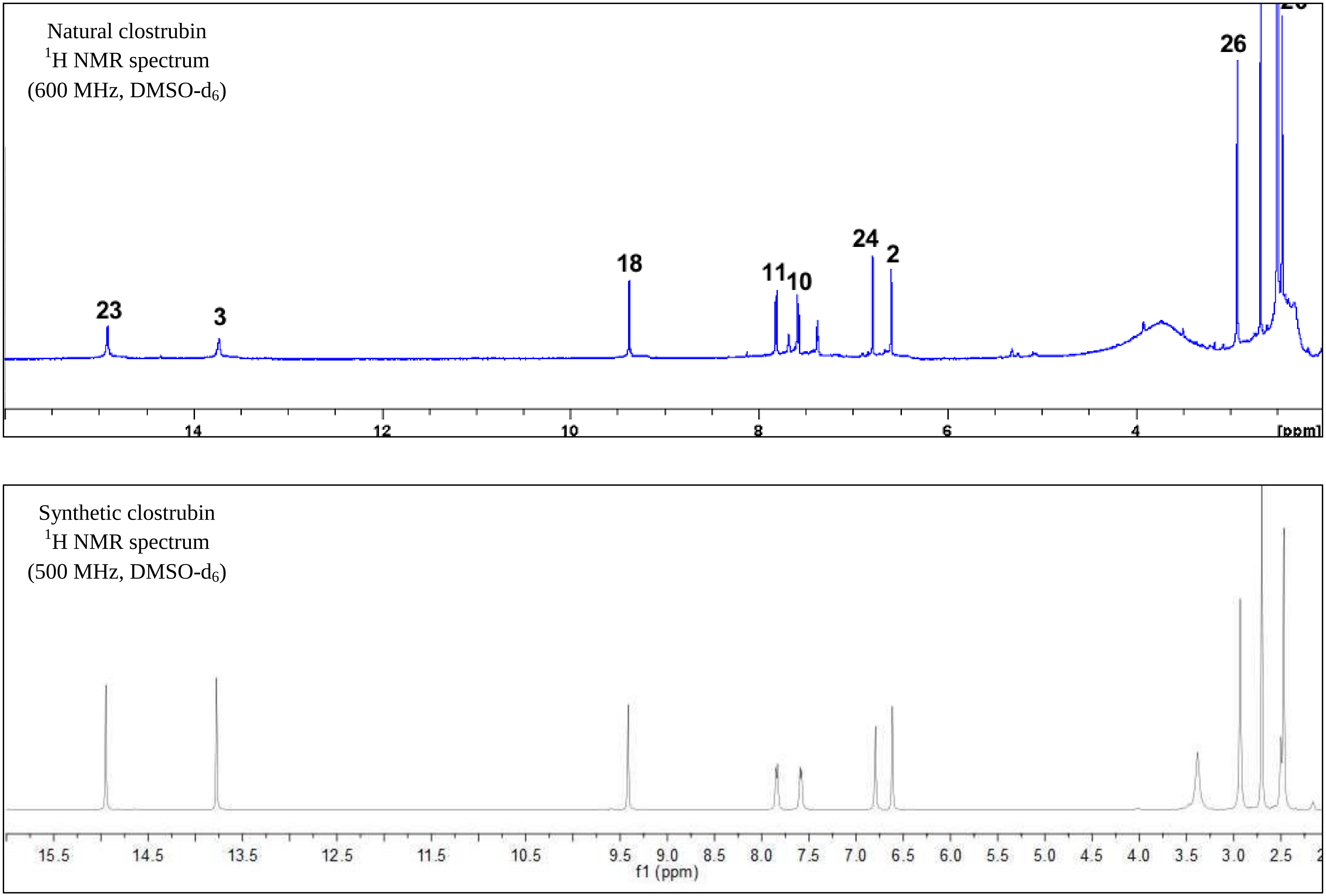

Supplementary Figure 32. Comparison of the ^{13}C NMR Spectra of Natural and Synthetic Clostrubin

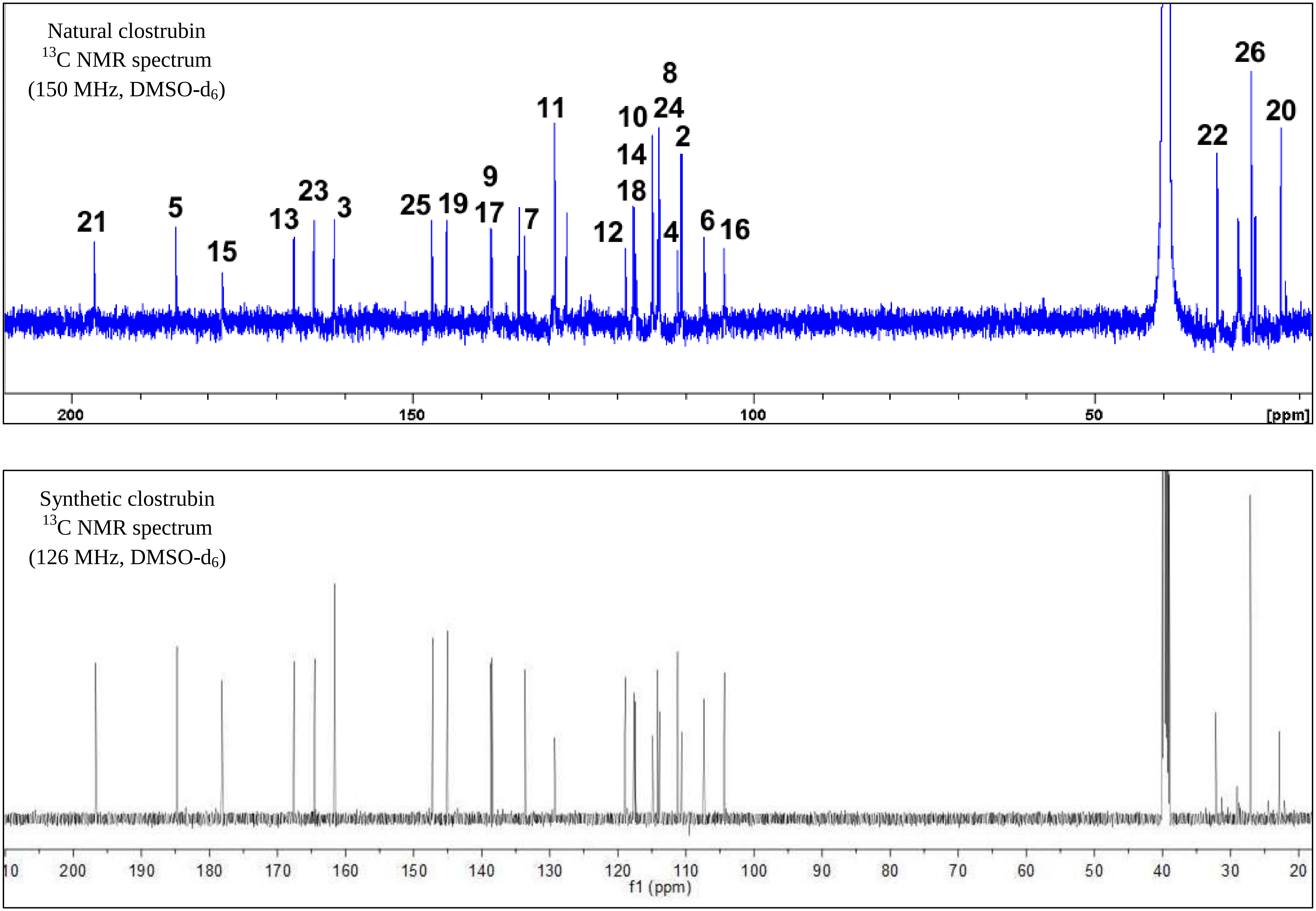

Supplementary Table 1. Comparison of the ^{1}H NMR (DMSO-d_6) data of natural and synthetic clostrubin

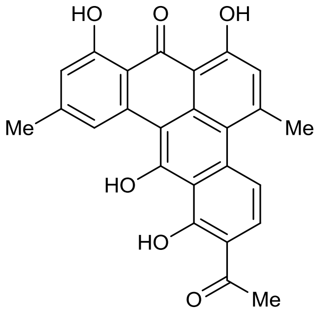

1: clostrubin

**Natural**<br>δ_H [ppm, mult, *J* (Hz)]<br>600 MHz	**Synthetic**<br>δ_H [ppm, mult, *J* (Hz)]<br>500 MHz	**Err**<br>(Natural−Synthetic)<br>$\Delta\delta_H$ (ppm)
14.88 1 H, s	14.93 1 H, s	−0.05
13.70 1 H, s	13.75 1 H, s	−0.05
9.38 1 H, s	9.39 1 H, s	−0.01
7.80 1 H, d, 9.0	7.82 1 H, d, 9.0	−0.02
7.60 1 H, d, 9.0	7.58 1 H, d, 9.0	0.02
6.80 1 H, s	6.79 1 H, s	0.01
6.60 1 H, s	6.60 1 H, s	0
2.90 3 H, s	2.93 3 H, s	−0.03
2.68 3 H, s	2.68 3 H, s	0
2.42 3 H, s	2.45 3 H, s	−0.03

Supplementary Table 2. Comparison of the ^{13}C NMR ($DMSO-d_6$) data of natural and synthetic clostrubin

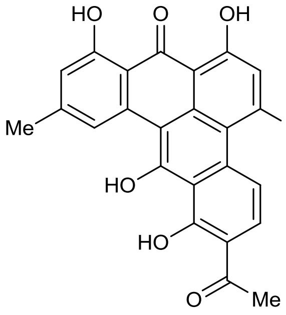

1: clostrubin

**Natural**<br>δ_C (ppm)<br>150 MHz	**Synthetic**<br>δ_C (ppm)<br>126 MHz	**Err**<br>(Natural−Synthetic)<br>$\Delta\delta_C$ (ppm)
196.8	196.7	0.1
184.7	184.8	−0.1
177.9	178.1	−0.2
167.4	167.6	−0.2
164.5	164.5	0
161.6	161.6	0
147.2	147.2	0
145.0	145.0	0
138.6	138.7	−0.1
138.4	138.5	−0.1
133.6	133.6	−0.1
129.2	129.3	−0.1
118.8	118.9	−0.1
117.6	117.6	−0.1
117.3	117.4	−0.1
114.9	114.9	−0.1
114.1	114.1	−0.1
114.0	113.9	0.1
111.2	111.2	0
110.6	110.6	0
107.3	107.3	0
104.3	104.4	−0.1

32.0	32.1	−0.1
27.0	27.1	−0.1
22.8	22.8	0

Supplementary Methods

I Experimental Procedures and Spectroscopic Data of Compounds

II Crystallographic Data of Compound 13

I Experimental Procedures and Spectroscopic Data of Compounds

General Procedures. All reactions were carried out under an argon atmosphere with dry solvents under anhydrous conditions, unless otherwise noted. Tetrahydrofuran (THF) was distilled immediately before use from sodium-benzophenone ketyl. Methylene chloride (CH_2Cl_2), *N*,*N*-dimethylformamide (DMF), triethylamine (Et_3N), diisopropylethylamine ($i Pr_2NEt$), and chlorotrimethylsilane (TMSCl) were distilled from calcium hydride and stored under an argon atmosphere. Methanol (MeOH) was distilled from magnesium and stored under an argon atmosphere. Reagents were purchased at the highest commercial quality and used without further purification, unless otherwise stated. Solvents for chromatography were used as supplied by Titan chemical. Reactions were monitored by thin layer chromatography (TLC) carried out on S-2 0.25 mm E. Merck silica gel plates (60F-254) using UV light as visualizing agent and aqueous ammonium cerium nitrate/ammonium molybdate or basic aqueous potassium permanganate as developing agent. E. Merck silica gel (60, particle size 0.040–0.063 mm) was used for flash column chromatography. Preparative thin layer chromatography separations were carried out on 0.25 or 0.50 mm E. Merck silica gel plates (60F-254). NMR spectra were recorded on Bruker AV-400 or Agilent 500/54/ASP instrument and calibrated by using residual undeuterated chloroform (δ_H = 7.26 ppm) and $CDCl_3$ (δ_C = 77.16 ppm), or undeuterated DMSO (δ_H = 2.50 ppm) and DMSO-d_6 (δ_C = 39.52 ppm), as internal references. The following abbreviations are used to designate multiplicities: s = singlet, d = doublet, t = triplet, q = quartet, m = multiplet, quint = quintet, br = broad. IR spectra were recorded on a Thermo Scientific Nicolet 380 FT-IR spectrometer. Melting points (m.p.) are uncorrected and were recorded on a Shanghai Jingke SGW X-4 apparatus. High-resolution mass spectra (HRMS) were recorded on a Bruker APEXIII 7.0 Tesla ESI-FT or a Waters Micromass GCT Premier EI mass spectrometer.

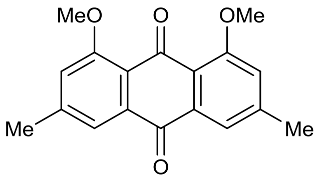

11

Anthraquinone 11: To a stirred solution of 2,6-dibromo-1,4-benzoquinone[1] **5** (12.0 g, 45.1 mmol) in THF (8.0 mL) was added Brassard diene[2] **8** (25.4 g, 136 mmol) at −78 °C. The reaction mixture was stirred at that temperature for 6 h before it was added silica gel (25.4 g) and allowed to warm to 22 °C. The resultant suspension was stirred at that temperature for 2 h before it was quenched with saturated aq. $NaHCO_3$ (100 mL). The mixture so obtained was extracted with CH_2Cl_2 (3 × 200 mL), and the combined organic phases were washed with brine (100 mL) and dried over anhydrous Na_2SO_4. After filtration and evaporation of the solvent under vacuum, the residue was dissolved in DMF (30 mL). To this solution were sequentially added anhydrous K_2CO_3 (18.8 g, 136 mmol) and MeI (16.2 g, 7.10 mL, 114 mmol) at 22 °C. The reaction mixture was allowed to stir at that temperature for 18 h before it was quenched with saturated aq. $NaHCO_3$ (100 mL). The resultant mixture was extracted with CH_2Cl_2 (3 × 200 mL). The combined organic phases were washed with water (100 mL) and brine (100 mL), dried over anhydrous Na_2SO_4, filtered, and concentrated under vacuum. The residue was subjected to flash column chromatography for purification using EtOAc/petroleum ether (1:2) and then EtOAc/CH_2Cl_2 (1:4) as eluent to give anthraquinone **11** (6.07 g, 45 % for the two steps) as an orange powder. **11**: R_f = 0.23 (silica, EtOAc:petroleum ether 1:1); IR (film): ν_{max} = 2923, 2848, 1668, 1600, 1460, 1330, 1306, 1239, 1162, 1032, 954, 756 cm^{-1}; ^{1}H NMR (500 MHz, $CDCl_3$): δ = 7.61 (s, 2 H), 7.06 (s, 2 H), 3.96 (s, 6 H), 2.44 (s, 6 H) ppm; ^{13}C NMR (126 MHz, $CDCl_3$): δ = 184.48, 182.40, 159.67, 144.84, 134.54, 121.58, 119.45, 118.72, 56.43, 22.08 ppm; HRMS (*m/z*): calcd for $[M + H]^+$ calcd for $C_{18}H_{17}O_4^+$ 297.1121, found 297.1125.

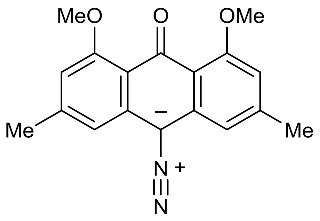

3

Diazoketone 3: To a suspension of anthraquinone **11** (1.15 g, 3.88 mmol) in AcOH (39 mL) was added a solution of $SnCl_2$•H_2O (5.28 g, 23.4 mmol) in aq. HCl (12 mL, 37 wt%) at 22 °C. The reaction mixture was stirred at that temperature for 30 min before it was diluted with water (100 mL). The resultant mixture was stirred for 10 min and extracted with CH_2Cl_2 (3 × 60 mL). The combined organic phases were washed with saturated aq. $NaHCO_3$ (2 × 80 mL) and brine (80 mL), dried over anhydrous Na_2SO_4, and filtered. After removal of the solvent under vacuum, the residue (crude anthranol **12**) was immediately dissolved in CH_2Cl_2 (39 mL). To this solution were sequentially added DBU (1.78 g, 1.75 mL, 11.7 mmol) and TsN_3 (853 mg, 880 μL, 4.32 mmol) at 22 °C. The reaction mixture was allowed to stir at that temperature for 20 h before it was diluted with CH_2Cl_2 (200 mL). The organic layer was sequentially washed with aq. HCl (100 mL, 1.0 M) and brine (100 mL), dried over anhydrous Na_2SO_4, and filtered. The solvent was evaporated under vacuum, and the residue was purified by flash column chromatography with EtOAc/CH_2Cl_2 (1:20 → 1:8) to give diazoketone **3** (1.06 g, 89 % for the two steps) as a purple-red foam. **3**: R_f = 0.33 (silica, EtOAc:CH_2Cl_2 1:8); IR (film): ν_{max} = 2948, 2842, 2055, 1646, 1606, 1256, 1041, 822 cm^{-1}; ^{1}H NMR (500 MHz, $CDCl_3$): δ = 6.51 (s, 2 H), 6.42 (s, 2 H), 3.89 (s, 6 H), 2.31 (s, 6 H) ppm; ^{13}C NMR (126 MHz, $CDCl_3$): δ = 179.71, 161.68, 143.38, 130.72, 117.80, 112.19, 108.97, 64.65, 55.98, 22.11 ppm; HRMS (*m/z*): calcd for $[M + H]^+$ calcd for $C_{18}H_{17}N_2O_3^+$ 309.1234, found 309.1233.

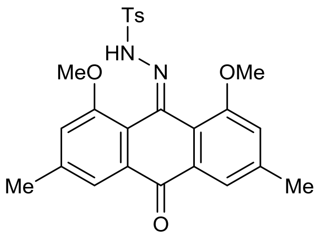

13

Tosylhydrazone 13: To a stirred solution of anthraquinone **11** (500 mg, 1.69 mmol) in ethanol (3.0 mL) were added $TsNHNH_2$ (315 mg, 1.70 mmol) and TsOH (29.3 mg, 0.170 mmol) at 22 ℃. The resultant mixture was warmed to 60 ℃ and stirred at that temperature for 2 h before it was cooled to 22 ℃ and quenched with saturated aq. $NaHCO_3$ (5 mL). The mixture so obtained was extracted with EtOAc (3 × 20 mL). The combined organic phases were washed with brine (10 mL) and dried over anhydrous Na_2SO_4. After filtration and evaporation of the solvent under vacuum, the residue was subjected to flash column chromatography for purification using EtOAc/petroleum ether (1:3) as eluent to give tosylhydrazone **13** (412 mg, 61 %) as a yellow solid. **13**: R_f = 0.53 (silica, EtOAc:petroleum ether 1:2); m.p. 194–195 ℃ (EtOAc:petroleum ether 1:1); IR (film): ν_{max} = 3168, 2942, 2842, 1670, 1602, 1462, 1348, 1321, 1297, 1168, 1030, 862 cm^{-1}; ^1H NMR (400 MHz, $CDCl_3$): δ = 9.36 (s, 1 H), 8.00 (d, *J* = 8.0 Hz, 2 H), 7.65 (s, 1 H), 7.49 (s, 1 H), 7.39 (d, *J* = 8.0 Hz, 2 H), 7.03 (s, 1 H), 6.96 (s, 1 H), 3.89 (s, 3 H), 3.77 (s, 3 H), 2.46 (s, 3 H), 2.44 (s, 3 H), 2.38 (s, 3 H) ppm; ^{13}C NMR (126 MHz, $CDCl_3$): δ = 184.69, 156.49, 153.87, 143.95, 142.61, 140.17, 140.10, 136.53, 134.71, 133.94, 129.50, 128.13, 125.35, 121.83, 119.31, 119.18, 118.38, 117.29, 56.91, 56.51, 21.73, 21.70 ppm; HRMS (*m/z*): calcd for $[M + H]^+$ calcd for $C_{25}H_{25}N_2O_5S^+$ 465.1479, found 465.1481. CCDC 1028256 contains the supplementary crystallographic data of **13**. These data can be obtained free of charge from The Cambridge Crystallographic Data Centre via www.ccdc.cam.ac.uk/data_request/cif.

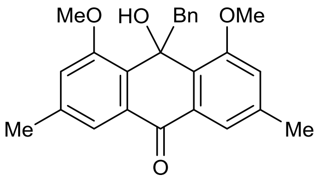

14

+

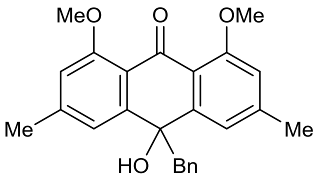

15

Tertiary Alcohols 14 and 15: To a stirred suspension of anthraquinone **11** (500 mg, 1.69 mmol) in THF (5.0 mL) was added BnMgBr (1.70 mL, 1.0 M in THF, 1.70 mmol) at 22 °C. The reaction mixture was stirred at that temperature for 15 min before it was quenched with saturated aq. NH_4Cl (5 mL) and extracted with EtOAc (3 × 15 mL). The combined organic phases were washed with brine (10 mL), dried over anhydrous Na_2SO_4, and filtered. The solvent was evaporated under vacuum, and the residue was purified by flash column chromatography with EtOAc/petroleum ether (2:5 → 1:1) as eluent to give tertiary alcohols **14** (210 mg, 32 %) as a pale yellow oil and **15** (262 mg, 40 %) as a dark yellow oil. **14**: R_f = 0.65 (silica, EtOAc:petroleum ether 1:1); IR (film): ν_{max} = 3523, 2937, 2840, 1664, 1609, 1455, 1341, 1282, 1078, 1035 cm^{-1}; ^1H NMR (400 MHz, $CDCl_3$): δ = 7.52 (s, 2 H), 7.10 (s, 2 H), 6.89 (t, *J* = 7.3 Hz, 1 H), 6.77 (t, *J* = 7.0 Hz, 2 H), 6.09 (d, *J* = 7.7 Hz, 2 H), 5.43 (s, 1 H), 4.09 (s, 6 H), 3.85 (s, 2 H), 2.42 (s, 6 H) ppm; ^{13}C NMR (126 MHz, $CDCl_3$): δ = 183.49, 157.10, 138.87, 136.62, 131.80, 130.38, 129.57, 127.33, 126.21, 119.74, 117.64, 75.18, 56.41, 45.69, 21.55 ppm; HRMS (*m/z*): calcd for $[M + Na]^+$ calcd for $C_{25}H_{24}O_4Na^+$ 411.1567, found 411.1569. **15**: R_f = 0.20 (silica, EtOAc:petroleum ether 1:1); IR (film): ν_{max} = 3440, 2940, 2840, 1662, 1607, 1459, 1304, 1251, 1046, 1031 cm^{-1}; ^1H NMR (400 MHz, $CDCl_3$): δ = 7.16 (dd, *J* = 7.2, 7.2 Hz, 1 H), 7.10 (dd, *J* = 7.3, 7.3 Hz, 2 H), 6.90 (s, 2 H), 6.72 (s, 2 H), 6.65 (d, *J* = 7.2 Hz, 2 H), 3.91 (s, 6 H), 3.01 (s, 2 H), 2.35 (s, 1 H), 2.31 (s, 6 H) ppm; ^{13}C NMR (101 MHz, $CDCl_3$): δ = 183.35, 158.75, 147.85, 143.45, 135.22, 130.93, 127.60, 127.02, 120.35, 117.47, 112.06, 73.89, 56.32, 55.12, 22.36 ppm; HRMS (*m/z*): $[M + H]^+$ calcd for $C_{25}H_{25}O_4^+$ 389.1747, found 389.1748.

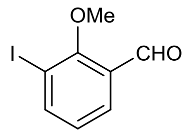

17

Methyl ether 17: To a stirred solution of phenol[3] **16** (2.50 g, 10.1 mmol) in DMF (10 mL) were sequentially added anhydrous K_2CO_3 (2.09 g, 15.1 mmol) and MeI (2.14 g, 940 μL, 15.1 mmol) at 22 °C. The reaction mixture was allowed to stir at that temperature for 24 h before it was diluted with water (40 mL). The resultant mixture was extracted with diethyl ether (3 × 60 mL). The combined organic phases were washed with water (40 mL) and brine (2 × 40 mL) and dried over anhydrous $MgSO_4$. After filtration and removal of the solvent under vacuum, methyl ether **17** (2.62 g, 99 %) was obtained as a pale yellow powder. **17**: R_f = 0.45 (silica, EtOAc:petroleum ether 1:20); IR (film): ν_{max} = 2962, 2936, 2856, 2741, 1701, 1581, 1456, 1417, 1384, 1242, 1109, 995, 799. 781, 691 cm^{-1}; ^{1}H NMR (500 MHz, $CDCl_3$): δ = 10.32 (s, 1 H), 8.03 (dd, *J* = 7.8, 1.7 Hz, 1 H), 7.82 (dd, *J* = 7.7, 1.7 Hz, 1 H), 7.01 (dd, *J* = 7.8, 7.7 Hz, 1 H), 3.95 (s, 3 H) ppm; ^{13}C NMR (126 MHz, $CDCl_3$): δ = 189.31, 162.66, 145.53, 130.28, 129.15, 126.51, 93.01, 64.01, 63.99 ppm; HRMS (*m/z*): $[M]^+$ calcd for $C_8H_7IO_2^+$ 261.9485, found 261.9491.

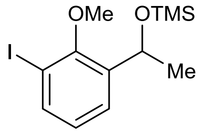

18

TMS ether 18: To a stirred solution of methyl ether **17** (2.62 g, 10.0 mmol) in THF (100 mL) was added MeMgBr (3.50 mL, 3.0 M in diethyl ether, 10.5 mmol) at 0 °C. The reaction mixture was stirred at that temperature for 10 min before HMPA (3.59 g, 3.50 mL, 20.0 mmol), Et_3N (1.53 g, 2.10 mL, 15.1 mmol), and TMSCl (1.61 g, 1.90 mL, 14.8 mmol) were sequentially added. The resultant mixture was stirred at 22 °C for 10 min and then quenched with saturated aq. NH_4Cl (50 mL). The mixture so obtained was extracted with EtOAc (3 × 80 mL). The combined organic phases were sequentially washed with water (80 mL) and brine (80 mL), dried over anhydrous Na_2SO_4, and filtered. The solvent was removed under vacuum, and the residue was purified by flash column chromatography with

Et_3N/petroleum ether (1:50) to give TMS ether **18** (3.28 g, 94 %) as a colorless oil. **18**: R_f = 0.33 (silica, petroleum ether); IR (film): ν_{max} = 2957, 1459, 1416, 1366, 1352, 1250, 1133, 1095, 1031, 1004, 959, 862, 841, 754 cm^{-1}; ^1H NMR (400 MHz, $CDCl_3$): δ = 7.67 (dd, J = 7.8, 1.5 Hz, 1 H), 7.51 (dd, J = 7.7, 1.5 Hz, 1 H), 6.87 (dd, J = 7.8, 7.7 Hz, 1 H), 5.18 (q, J = 6.3 Hz, 1 H), 3.83 (s, 3 H), 1.41 (d, J = 6.3 Hz, 3 H), 0.08 (s, 9 H) ppm; ^{13}C NMR (126 MHz, $CDCl_3$): δ = 155.74, 141.47, 138.21, 127.52, 126.51, 91.53, 65.78, 61.45, 26.58, 0.23 ppm; HRMS (*m/z*): $[M]^+$ calcd for $C_{12}H_{19}IO_2Si^+$ 350.0194, found 350.0199.

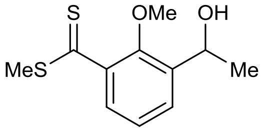

20

Benzylic alcohol 20: To a stirred solution of TMS ether **18** (1.12 g, 3.20 mmol) in THF (30 mL) was added EtMgBr (1.60 mL, 3.0 M in diethyl ether, 4.80 mmol) at 22 °C. The resultant mixture was warmed to 50 °C and stirred at that temperature for 1 h before CS_2 (7.56 g, 6.00 mL, 99.5 mmol) was added. The reaction mixture was heated to 70 °C and allowed to stir at that temperature for 3 h before it was cooled to 22 °C. MeI (1.82 g, 800 μL, 12.8 mmol) was added at 22 °C, and the resultant mixture was allowed to stir at that temperature for 8 h before it was quenched with aq. HCl (30 mL, 1.0 M) and extracted with EtOAc (3 × 40 mL). The combined organic phases were sequentially washed with saturated aq. $NaHCO_3$ (50 mL) and brine (50 mL), dried over anhydrous Na_2SO_4, and filtered. After removal of the solvent under vacuum, the residue was purified by flash column chromatography with EtOAc/petroleum ether (1:6) to give benzylic alcohol **20** (534 mg, 67 %) as a bright orange oil. **20**: R_f = 0.35 (silica, EtOAc:petroleum ether 1:4); IR (film): ν_{max} = 3401, 2971, 2935, 2826, 1584, 1459, 1418, 1222, 1069, 1012, 959, 887, 799 cm^{-1}; ^1H NMR (400 MHz, $CDCl_3$): δ = 7.51 (dd, J = 7.7, 1.7 Hz, 1 H), 7.31 (dd, J = 7.7, 1.7 Hz, 1 H), 7.13 (dd, J = 7.7, 7.7 Hz, 1 H), 5.24 (dq, J = 6.5, 4.8 Hz, 1 H), 3.74 (s, 3 H), 2.78 (s, 3 H), 2.48–2.40 (m, 1 H), 1.51 (d, J = 6.5 Hz, 3 H); ^{13}C NMR (101 MHz, $CDCl_3$): δ = 152.55, 140.87, 139.35, 128.61, 128.45, 124.19, 65.12, 62.34, 24.17, 21.16 ppm; HRMS (*m/z*): $[M + Na]^+$ calcd for $C_{11}H_{14}O_2S_2Na^+$ 265.0327, found 265.0333.

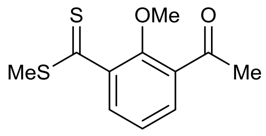

21

Ketodithioester 21: To a stirred solution of benzylic alcohol **20** (453 mg, 1.87 mmol) in CH_2Cl_2 (10 mL) was added Dess−Martin periodinane (951 mg, 2.24 mmol) at 22 °C. The resultant mixture was stirred at that temperature for 1 min before it was diluted with EtOAc (50 mL) and quenched with saturated aq. $NaHSO_3$ (20 mL). The aqueous layer was separated. The organic phase was washed with saturated aq. $NaHCO_3$ (30 mL) and brine (30 mL), dried over anhydrous Na_2SO_4, and filtered. The solvent was evaporated under vacuum. The residue so obtained was subjected to flash column chromatography for purification using EtOAc/petroleum ether (1:15) as eluent to give ketodithioester **21** (371 mg, 83 %) as a bright orange oil. **21**: R_f = 0.55 (silica, EtOAc:petroleum ether 1:4); IR (film): ν_{max} = 3067, 2997, 2939, 2842, 1687, 1578, 1460, 1410, 1355, 1281, 1255, 1063, 919, 799 cm^{-1}; ^1H NMR (400 MHz, $CDCl_3$): δ = 7.63 (dd, *J* = 7.7, 1.8 Hz, 1 H), 7.49 (dd, *J* = 7.6, 1.8 Hz, 1 H), 7.16 (dd, *J* = 7.7, 7.6 Hz, 1 H), 3.77 (s, 3 H), 2.79 (s, 3 H), 2.64 (s, 3 H) ppm; ^{13}C NMR (101 MHz, $CDCl_3$): δ = 200.06, 154.66, 141.74, 134.12, 132.54, 131.38, 123.86, 63.43, 30.68, 21.10 ppm; HRMS (*m/z*): $[M + Na]^+$ calcd for $C_{11}H_{12}O_2S_2Na^+$ 263.0171, found 263.0175.

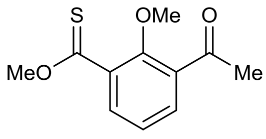

4

Thioester 4: To a stirred solution of ketodithioester **21** (200 mg, 0.832 mmol) in MeOH (8.0 mL) was added MeONa (450 mg, 8.33 mmol) at 22 °C. The resultant mixture was warmed to 50 °C and allowed to stir at that temperature for 4 h before it was cooled to 22 °C and quenched with water (10 mL). The mixture so obtained was extracted with EtOAc (3 × 20 mL), and the combined organic phases were washed with brine (2 × 20 mL) and filtered. After removal of solvent under vacuum, the residue was purified by flash column chromatography with EtOAc/petroleum ether (1:20) to give thioester **4** (124 mg, 66 %) as a bright yellow oil. **4**: R_f = 0.42 (silica, EtOAc:petroleum ether 1:8); IR (film): ν_{max} = 2995,

2941, 2843, 1686, 1579, 1462, 1438, 1414, 1238, 1068, 1001, 804, 763 cm^{-1}; ^{1}H NMR (500 MHz, $CDCl_3$): δ = 7.79 (dd, *J* = 7.6, 1.6 Hz, 1 H), 7.66 (dd, *J* = 7.8, 1.6 Hz, 1 H), 7.15 (dd, *J* = 7.8, 7.6 Hz, 1 H), 4.31 (s, 3 H), 3.80 (s, 3 H), 2.63 (s, 3 H) ppm; ^{13}C NMR (101 MHz, $CDCl_3$): δ = 213.56, 200.27, 155.76, 136.06, 135.22, 134.22, 132.20, 123.72, 63.05, 59.78, 30.85 ppm; HRMS (*m/z*): $[M + Na]^+$ calcd for $C_{11}H_{12}O_3SNa^+$ 247.0399, found 247.0401.

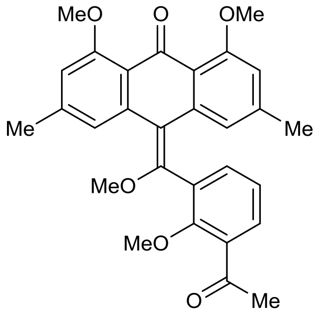

2

Tetrasubstituted olefin 2: To a stirred solution of thioester **4** (90.0 mg, 0.401 mmol) and $Rh_2(OAc)_4$ (8.9 mg, 0.020 mmol) in toluene (6.0 mL) was added diazoketone **3** (185 mg, 0.600 mmol) in five portions at 50 °C over a period of 40 min. The resultant mixture was stirred at that temperature for 15 min before it was cooled to 22 °C and Cu powder (200 mg, 3.15 mmol) was added. The mixture so obtained was heated to 110 °C and stirred at that temperature for 1.5 h before it was cooled to 22 °C. The resultant mixture was directly subjected to flash column chromatography for purification using EtOAc/CH_2Cl_2 (1:20 → 1:6) as eluent to give tetrasubstituted olefin **2** (162 mg, 85 %) as a pale yellow foam. **2**: R_f = 0.33 (silica, diethyl ether:CH_2Cl_2 1:8); IR (film): ν_{max} = 3059, 2962, 2940, 2840, 1671, 1602, 1461, 1411, 1261, 1063, 1044, 803 cm^{-1}; ^{1}H NMR (500 MHz, $CDCl_3$): δ = 7.61 (s, 1 H), 7.58 (dd, *J* = 7.6, 1.7 Hz, 1 H), 7.24 (br s, 1 H), 7.03 (dd, *J* = 7.6 , 7.6 Hz, 1 H), 6.71 (s, 1 H), 6.47 (s, 1 H), 6.13 (s, 1 H), 3.91 (s, 3 H), 3.84 (s, 3 H), 3.82 (br s, 3 H), 3.37 (s, 3 H), 2.49 (br s, 3 H), 2.42 (s, 3 H), 1.93 (s, 3 H) ppm; ^{13}C NMR (126 MHz, $CDCl_3$): δ = 200.67, 185.26, 157.89, 157.84, 152.66, 141.80, 140.91, 139.67, 137.89, 136.68, 133.82, 130.93, 127.40, 123.53, 122.15, 121.91, 121.66, 120.85, 116.20, 111.11, 110.24, 61.32, 56.67, 56.13, 55.97, 30.92, 22.45, 21.97 ppm; HRMS (*m/z*): $[M + H]^+$ calcd for $C_{29}H_{29}O_6^+$ 473.1959, found 473.1954.

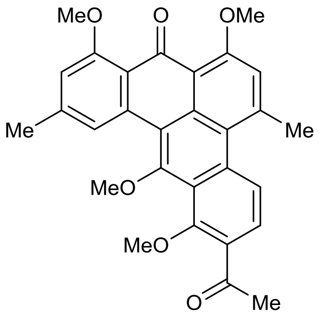

24

Tetramethyl clostrubin 24: A solution of tetrasubstituted olefin **2** (195 mg, 0.412 mmol) in $CHCl_3$ (410 mL) in a quartz vessel was irradiated with UV light (λ = 365 nm, from four 8-Watt Hg lamps) at 22 °C for 8 h. The volatile was then removed under vacuum, and the residue was subjected to HPLC (Waters 2535Q, SunFire Prep C18 OBD column) using acetonitrile/water (70:30, with 0.07 vol% of AcOH, 10 mL/min) as eluent to give tetramethyl clostrubin **24** (t_R = 16.13 min). After removal of the solvent under vacuum, **24** (107 mg, 55 %) was obtained as a pale yellow foam. **24**: R_f = 0.33 (silica, EtOAc:CH_2Cl_2 1:8); IR (film): ν_{max} = 2963, 2934, 2845, 1660, 1604, 1587, 1461, 1324, 1305, 1262, 1084, 1023, 989, 801 cm^{-1}; ^1H NMR (500 MHz, $CDCl_3$): δ = 8.28 (d, J = 8.8 Hz, 1 H), 8.20 (s, 1 H), 7.78 (d, J = 8.8 Hz, 1 H), 7.19 (s, 1 H), 6.84 (s, 1 H), 4.09 (s, 3 H), 4.01 (s, 3 H), 4.00 (s, 3 H), 3.88 (s, 3 H), 3.04 (s, 3 H), 2.79 (s, 3 H), 2.47 (s, 3 H) ppm; ^{13}C NMR (101 MHz, $CDCl_3$): δ = 201.17, 184.32, 158.90, 158.60, 157.62, 156.86, 142.95, 142.16, 137.34, 136.36, 132.67, 131.12, 126.52, 123.26, 122.14, 121.34, 121.07, 120.54, 117.32, 116.99, 116.03, 111.95, 64.65, 62.54, 56.52, 56.22, 31.23, 27.38, 22.63 ppm; HRMS (m/z): $[M + H]^+$ calcd for $C_{29}H_{27}O_6^+$ 471.1802, found 471.1810.

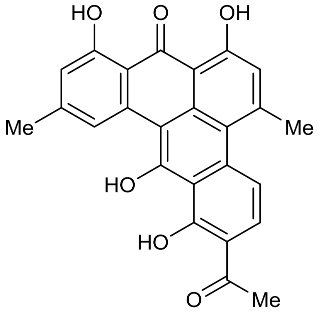

1: clostrubin

Clostrubin (1): To a stirred solution of tetramethyl clostrubin **24** (59.4 mg, 0.126 mmol) in AcOH (19.0 mL) was added aq. HBr (6.3 mL, 48 wt%) at 22 °C. The resultant mixture was sealed in a vessel and heated to 120 °C. After stirring at that temperature for 10 h, the reaction mixture was cooled to 22 °C

and diluted with water (40 mL) and neutralized with aq. NaOH (75 mL, 5.0 M) followed by saturated aq. $NaHCO_3$ (50 mL). The mixture so obtained was extracted with EtOAc (3 × 60 mL). The combined organic phases were washed with brine (80 mL) and dried over anhydrous Na_2SO_4. After filtration and evaporation of the solvent under vacuum, clostrubin (**1**, 49.8 mg, 95 %) was obtained as an orange powder. **1**: R_f = 0.42 (silica, EtOAc:CH_2Cl_2 1:8); IR (film): ν_{max} = 2958, 2925, 2852, 1583, 1465, 1407, 1377, 1191, 1088 cm^{-1}; ^1H NMR (500 MHz, DMSO-d_6): δ = 14.93 (s, 1 H), 13.75 (s, 1 H), 9.39 (s, 1 H), 7.82 (d, *J* = 9.0 Hz, 1 H), 7.58 (d, *J* = 9.0 Hz, 1 H), 6.79 (s, 1 H), 6.60 (s, 1 H), 2.93 (s, 3 H), 2.68 (s, 3 H), 2.45 (s, 3 H) ppm; ^{13}C NMR (126 MHz, DMSO-d_6): δ = 196.70, 184.75, 178.14, 167.57, 164.54, 161.59, 147.20, 145.04, 138.67, 138.47, 133.62, 129.27, 118.89, 117.62, 117.42, 114.89, 114.16, 113.91, 111.22, 110.64, 107.32, 104.35, 32.14, 27.09, 22.81 ppm; HRMS (*m/z*): $[M - H]^-$ calcd for $C_{25}H_{17}O_6^-$ 413.1031, found 413.1031.

II Crystallographic Data of Compounds 13

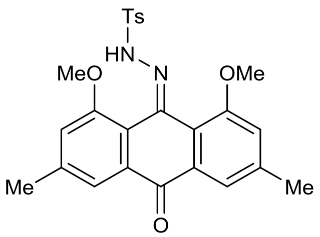

13

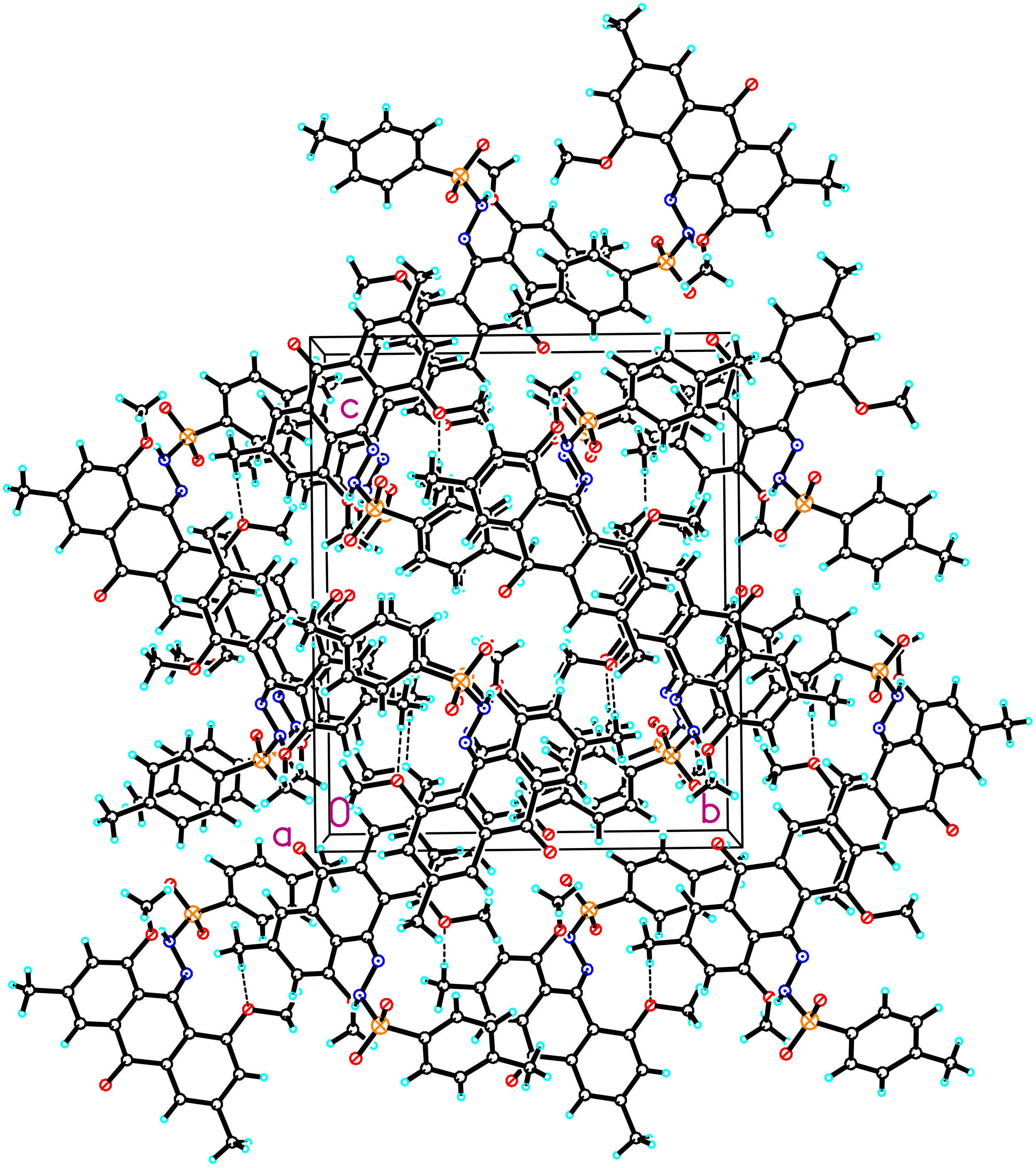

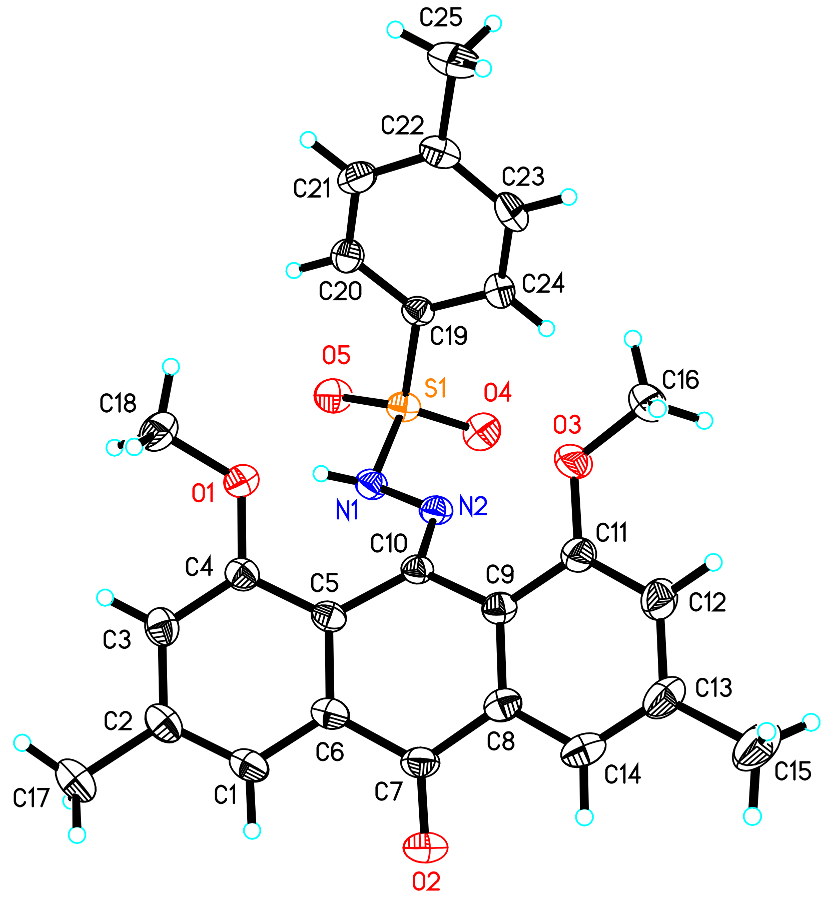

Crystal data

$C_{25}H_{24}N_2O_5S$	D_x = 1.352 Mg m^{-3}
M_r = 464.52	Mo $K\alpha$ radiation, λ = 0.71073 Å
Orthorhombic, $P2_12_12_1$	Cell parameters from 3872 reflections
a = 8.4096 (6) Å	θ = 4.5–45.6 °
b = 15.0102 (12) Å	μ = 0.18 mm^{-1}
c = 18.0794 (14) Å	T = 293 K
V = 2282.2 (3) Å^3	Prismatic, yellow
Z = 4	0.21 × 0.17 × 0.12 mm
F(000) = 976	

Data collection

CCD area detector diffractometer	3926 reflections with $I > 2\sigma(I)$
phi and ω scans	R_{int} = 0.036
Absorption correction: multi-scan *SADABS*	θ_{max} = 26.0 °, θ_{min} = 1.8 °
T_{min} = 0.660, T_{max} = 0.746	h = −10→10
13956 measured reflections	k = −18→11
4505 independent reflections	l = −22→22

Refinement

Refinement on F^2	Hydrogen site location: mixed

Least-squares matrix: full	H atoms treated by a mixture of independent and constrained refinement
$R[F^2 > 2\sigma(F^2)] = 0.040$	$w = 1/[\sigma^2(F_o^2) + (0.0619P)^2]$ where $P = (F_o^2 + 2F_c^2)/3$
$wR(F^2) = 0.102$	$(\Delta/\sigma)_{max} = 0.001$
$S = 1.03$	$\Delta\rho_{max} = 0.22$ e Å^{-3}
4505 reflections	$\Delta\rho_{min} = -0.17$ e Å^{-3}
307 parameters	Absolute structure: Flack x determined using 1512 quotients [(I+)-(I-)]/[(I+)+(I-)] (Parsons and Flack (2004), Acta Cryst. A60, s61).
0 restraints	Flack parameter: 0.04 (4)

Special details

Geometry. All esds (except the esd in the dihedral angle between two l.s. planes) are estimated using the full covariance matrix. The cell esds are taken into account individually in the estimation of esds in distances, angles and torsion angles; correlations between esds in cell parameters are only used when they are defined by crystal symmetry. An approximate (isotropic) treatment of cell esds is used for estimating esds involving l.s. planes.

Fractional atomic coordinates and isotropic or equivalent isotropic displacement parameters (Å^2)

	x	y	z	U_{iso}*/U_{eq}
S1	−0.08194 (8)	0.64978 (5)	0.83329 (4)	0.0402 (2)
N1	0.0560 (3)	0.59861 (16)	0.78303 (13)	0.0404 (6)
N2	0.0879 (3)	0.64192 (15)	0.71764 (12)	0.0385 (5)
O1	0.3651 (2)	0.57418 (15)	0.81274 (11)	0.0485 (5)
O2	0.2863 (4)	0.4514 (2)	0.50231 (13)	0.0752 (8)
O3	0.1483 (3)	0.79858 (14)	0.64569 (11)	0.0607 (7)
O4	−0.2126 (2)	0.66787 (17)	0.78620 (12)	0.0557 (6)
O5	−0.1010 (3)	0.59266 (14)	0.89564 (11)	0.0535 (6)
C1	0.4237 (4)	0.4012 (2)	0.63686 (18)	0.0491 (7)
H1	0.4380	0.3639	0.5964	0.059*
C2	0.4872 (4)	0.3773 (2)	0.7043 (2)	0.0486 (8)
C3	0.4686 (4)	0.4361 (2)	0.76306 (18)	0.0473 (8)
H3	0.5153	0.4225	0.8083	0.057*
C4	0.3820 (3)	0.51458 (19)	0.75602 (16)	0.0403 (7)
C5	0.3090 (3)	0.53668 (19)	0.68865 (15)	0.0375 (6)
C6	0.3393 (4)	0.4795 (2)	0.62815 (17)	0.0438 (7)
C7	0.2848 (4)	0.5049 (2)	0.55296 (17)	0.0465 (7)
C8	0.2418 (3)	0.5996 (2)	0.54042 (16)	0.0434 (7)
C9	0.2086 (3)	0.6545 (2)	0.60105 (14)	0.0389 (6)
C10	0.2029 (3)	0.61375 (18)	0.67607 (14)	0.0353 (6)
C11	0.1747 (4)	0.7447 (2)	0.58660 (16)	0.0457 (7)
C12	0.1732 (5)	0.7753 (2)	0.51405 (17)	0.0565 (9)
H12	0.1507	0.8351	0.5053	0.068*
C13	0.2039 (5)	0.7204 (3)	0.45425 (17)	0.0587 (9)
C14	0.2378 (4)	0.6318 (2)	0.46840 (17)	0.0525 (9)
H14	0.2581	0.5933	0.4292	0.063*

C15	0.2051 (7)	0.7575 (3)	0.37636 (18)	0.0873 (15)
H15A	0.1111	0.7926	0.3685	0.131*
H15B	0.2075	0.7093	0.3415	0.131*
H15C	0.2974	0.7942	0.3697	0.131*
C16	0.1372 (5)	0.8917 (2)	0.6334 (2)	0.0619 (10)
H16A	0.2322	0.9124	0.6095	0.093*
H16B	0.1248	0.9218	0.6799	0.093*
H16C	0.0470	0.9041	0.6025	0.093*
C17	0.5731 (5)	0.2901 (2)	0.7140 (2)	0.0635 (9)
H17A	0.4970	0.2431	0.7206	0.095*
H17B	0.6409	0.2934	0.7566	0.095*
H17C	0.6362	0.2782	0.6708	0.095*
C18	0.4702 (4)	0.5694 (3)	0.87442 (17)	0.0552 (9)
H18A	0.4513	0.5152	0.9011	0.083*
H18B	0.4519	0.6194	0.9064	0.083*
H18C	0.5782	0.5705	0.8573	0.083*
C19	0.0004 (3)	0.75145 (19)	0.86365 (15)	0.0376 (6)
C20	0.0882 (4)	0.7532 (2)	0.92847 (17)	0.0490 (7)
H20	0.1016	0.7015	0.9562	0.059*
C21	0.1552 (5)	0.8317 (2)	0.95138 (18)	0.0565 (9)
H21	0.2154	0.8326	0.9945	0.068*
C22	0.1350 (4)	0.9097 (2)	0.91158 (19)	0.0516 (8)
C23	0.0488 (4)	0.9059 (2)	0.84589 (19)	0.0538 (8)
H23	0.0367	0.9572	0.8177	0.065*
C24	−0.0190 (4)	0.8275 (2)	0.82200 (18)	0.0459 (7)
H24	−0.0772	0.8260	0.7783	0.055*
C25	0.2060 (6)	0.9966 (3)	0.9372 (3)	0.0814 (13)
H25A	0.1222	1.0371	0.9504	0.122*
H25B	0.2684	1.0220	0.8981	0.122*
H25C	0.2725	0.9861	0.9795	0.122*
H1A	0.138 (4)	0.585 (2)	0.8084 (16)	0.040 (8)*

Atomic displacement parameters (Å^2)

	U^{11}	U^{22}	U^{33}	U^{12}	U^{13}	U^{23}
S1	0.0405 (4)	0.0409 (4)	0.0393 (3)	−0.0043 (3)	0.0088 (3)	−0.0024 (3)
N1	0.0462 (14)	0.0347 (13)	0.0404 (13)	0.0003 (12)	0.0030 (11)	−0.0002 (11)
N2	0.0483 (13)	0.0325 (12)	0.0349 (11)	−0.0034 (12)	0.0045 (10)	−0.0020 (10)
O1	0.0522 (12)	0.0496 (12)	0.0437 (11)	0.0039 (10)	−0.0084 (9)	−0.0078 (10)
O2	0.1008 (19)	0.0696 (18)	0.0551 (14)	0.0246 (17)	−0.0104 (14)	−0.0281 (14)
O3	0.104 (2)	0.0345 (12)	0.0433 (12)	0.0071 (12)	0.0076 (12)	0.0026 (10)
O4	0.0417 (11)	0.0687 (15)	0.0566 (12)	−0.0030 (12)	0.0018 (10)	−0.0011 (12)
O5	0.0661 (14)	0.0467 (12)	0.0476 (11)	−0.0116 (12)	0.0123 (11)	0.0040 (11)
C1	0.0463 (16)	0.0403 (17)	0.0605 (19)	0.0057 (15)	0.0038 (15)	−0.0113 (15)
C2	0.0392 (15)	0.0397 (17)	0.067 (2)	0.0035 (13)	0.0068 (15)	0.0010 (16)
C3	0.0398 (15)	0.0468 (18)	0.0552 (18)	0.0019 (14)	−0.0026 (14)	0.0036 (16)

C4	0.0379 (15)	0.0376 (15)	0.0453 (15)	−0.0016 (13)	0.0005 (12)	−0.0019 (14)
C5	0.0383 (14)	0.0320 (14)	0.0424 (15)	−0.0033 (12)	0.0043 (12)	−0.0025 (13)
C6	0.0416 (16)	0.0404 (16)	0.0493 (16)	0.0003 (13)	0.0034 (13)	−0.0098 (14)
C7	0.0470 (17)	0.0494 (18)	0.0430 (16)	0.0049 (15)	0.0028 (14)	−0.0121 (16)
C8	0.0412 (15)	0.0501 (18)	0.0390 (15)	−0.0034 (14)	0.0024 (12)	−0.0049 (15)
C9	0.0408 (14)	0.0391 (15)	0.0368 (14)	−0.0042 (13)	0.0032 (12)	−0.0017 (13)
C10	0.0387 (13)	0.0311 (13)	0.0360 (13)	−0.0030 (12)	−0.0004 (12)	−0.0037 (12)
C11	0.0562 (19)	0.0422 (17)	0.0388 (15)	−0.0013 (15)	0.0006 (14)	0.0009 (14)
C12	0.076 (2)	0.045 (2)	0.0477 (17)	−0.0101 (18)	−0.0059 (17)	0.0108 (16)
C13	0.074 (2)	0.065 (2)	0.0374 (16)	−0.0218 (19)	−0.0041 (16)	0.0052 (17)
C14	0.0570 (19)	0.064 (2)	0.0366 (15)	−0.0113 (17)	0.0058 (14)	−0.0098 (16)
C15	0.134 (4)	0.086 (3)	0.0420 (18)	−0.028 (3)	−0.009 (2)	0.009 (2)
C16	0.088 (3)	0.0356 (17)	0.062 (2)	0.0022 (18)	−0.0029 (19)	0.0025 (17)
C17	0.056 (2)	0.0471 (18)	0.087 (2)	0.0141 (17)	0.006 (2)	0.0000 (19)
C18	0.0551 (19)	0.061 (2)	0.0492 (18)	−0.0103 (16)	−0.0107 (15)	0.0004 (17)
C19	0.0395 (15)	0.0376 (15)	0.0357 (14)	0.0011 (12)	0.0080 (12)	−0.0021 (13)
C20	0.0641 (19)	0.0439 (17)	0.0389 (14)	0.0008 (17)	0.0007 (15)	0.0047 (14)
C21	0.070 (2)	0.055 (2)	0.0443 (16)	−0.0052 (17)	−0.0088 (16)	−0.0092 (17)
C22	0.0577 (19)	0.0397 (17)	0.0573 (19)	−0.0009 (15)	0.0098 (15)	−0.0090 (16)
C23	0.0604 (19)	0.0359 (16)	0.065 (2)	0.0096 (15)	0.0045 (17)	0.0085 (16)
C24	0.0496 (16)	0.0414 (16)	0.0467 (17)	0.0051 (13)	−0.0029 (14)	0.0041 (15)
C25	0.095 (3)	0.051 (2)	0.098 (3)	−0.007 (2)	0.002 (3)	−0.025 (2)

Geometric parameters (Å, º)

S1—O4	1.416 (2)	C12—H12	0.9300
S1—O5	1.425 (2)	C13—C14	1.384 (5)
S1—N1	1.662 (3)	C13—C15	1.515 (5)
S1—C19	1.764 (3)	C14—H14	0.9300
N1—N2	1.376 (3)	C15—H15A	0.9600
N1—H1A	0.85 (3)	C15—H15B	0.9600
N2—C10	1.296 (4)	C15—H15C	0.9600
O1—C4	1.368 (3)	C16—H16A	0.9600
O1—C18	1.425 (4)	C16—H16B	0.9600
O2—C7	1.218 (4)	C16—H16C	0.9600
O3—C11	1.358 (4)	C17—H17A	0.9600
O3—C16	1.419 (4)	C17—H17B	0.9600
C1—C2	1.378 (5)	C17—H17C	0.9600
C1—C6	1.382 (4)	C18—H18A	0.9600
C1—H1	0.9300	C18—H18B	0.9600
C2—C3	1.389 (5)	C18—H18C	0.9600
C2—C17	1.506 (4)	C19—C24	1.378 (4)
C3—C4	1.391 (4)	C19—C20	1.385 (4)
C3—H3	0.9300	C20—C21	1.370 (5)
C4—C5	1.404 (4)	C20—H20	0.9300
C5—C6	1.413 (4)	C21—C22	1.384 (5)

C5—C10	1.479 (4)	C21—H21	0.9300
C6—C7	1.484 (4)	C22—C23	1.393 (5)
C7—C8	1.484 (5)	C22—C25	1.508 (5)
C8—C14	1.389 (4)	C23—C24	1.376 (5)
C8—C9	1.399 (4)	C23—H23	0.9300
C9—C11	1.408 (4)	C24—H24	0.9300
C9—C10	1.489 (4)	C25—H25A	0.9600
C11—C12	1.390 (4)	C25—H25B	0.9600
C12—C13	1.384 (5)	C25—H25C	0.9600
O4—S1—O5	120.24 (14)	C13—C14—C8	120.8 (3)
O4—S1—N1	107.54 (13)	C13—C14—H14	119.6
O5—S1—N1	103.46 (13)	C8—C14—H14	119.6
O4—S1—C19	109.02 (15)	C13—C15—H15A	109.5
O5—S1—C19	108.57 (13)	C13—C15—H15B	109.5
N1—S1—C19	107.21 (13)	H15A—C15—H15B	109.5
N2—N1—S1	112.8 (2)	C13—C15—H15C	109.5
N2—N1—H1A	115 (2)	H15A—C15—H15C	109.5
S1—N1—H1A	112.4 (19)	H15B—C15—H15C	109.5
C10—N2—N1	119.3 (2)	O3—C16—H16A	109.5
C4—O1—C18	119.3 (2)	O3—C16—H16B	109.5
C11—O3—C16	118.3 (2)	H16A—C16—H16B	109.5
C2—C1—C6	121.4 (3)	O3—C16—H16C	109.5
C2—C1—H1	119.3	H16A—C16—H16C	109.5
C6—C1—H1	119.3	H16B—C16—H16C	109.5
C1—C2—C3	117.9 (3)	C2—C17—H17A	109.5
C1—C2—C17	121.0 (3)	C2—C17—H17B	109.5
C3—C2—C17	121.2 (3)	H17A—C17—H17B	109.5
C2—C3—C4	121.8 (3)	C2—C17—H17C	109.5
C2—C3—H3	119.1	H17A—C17—H17C	109.5
C4—C3—H3	119.1	H17B—C17—H17C	109.5
O1—C4—C3	122.7 (3)	O1—C18—H18A	109.5
O1—C4—C5	116.8 (2)	O1—C18—H18B	109.5
C3—C4—C5	120.6 (3)	H18A—C18—H18B	109.5
C4—C5—C6	116.7 (3)	O1—C18—H18C	109.5
C4—C5—C10	125.6 (2)	H18A—C18—H18C	109.5
C6—C5—C10	117.7 (2)	H18B—C18—H18C	109.5
C1—C6—C5	121.4 (3)	C24—C19—C20	120.7 (3)
C1—C6—C7	118.8 (3)	C24—C19—S1	120.0 (2)
C5—C6—C7	119.8 (3)	C20—C19—S1	119.3 (2)
O2—C7—C8	121.3 (3)	C21—C20—C19	119.4 (3)
O2—C7—C6	121.0 (3)	C21—C20—H20	120.3
C8—C7—C6	117.5 (3)	C19—C20—H20	120.3
C14—C8—C9	121.6 (3)	C20—C21—C22	121.3 (3)
C14—C8—C7	118.8 (3)	C20—C21—H21	119.3
C9—C8—C7	119.5 (3)	C22—C21—H21	119.3

C8—C9—C11	117.5 (3)	C21—C22—C23	118.2 (3)
C8—C9—C10	118.6 (3)	C21—C22—C25	121.6 (3)
C11—C9—C10	123.9 (2)	C23—C22—C25	120.2 (3)
N2—C10—C5	128.1 (2)	C24—C23—C22	121.2 (3)
N2—C10—C9	114.7 (2)	C24—C23—H23	119.4
C5—C10—C9	116.2 (2)	C22—C23—H23	119.4
O3—C11—C12	122.9 (3)	C23—C24—C19	119.2 (3)
O3—C11—C9	117.4 (2)	C23—C24—H24	120.4
C12—C11—C9	119.7 (3)	C19—C24—H24	120.4
C13—C12—C11	122.6 (3)	C22—C25—H25A	109.5
C13—C12—H12	118.7	C22—C25—H25B	109.5
C11—C12—H12	118.7	H25A—C25—H25B	109.5
C14—C13—C12	117.8 (3)	C22—C25—H25C	109.5
C14—C13—C15	121.6 (3)	H25A—C25—H25C	109.5
C12—C13—C15	120.5 (4)	H25B—C25—H25C	109.5

O4—S1—N1—N2	49.3 (2)	C6—C5—C10—N2	135.2 (3)
O5—S1—N1—N2	177.56 (19)	C4—C5—C10—C9	147.6 (3)
C19—S1—N1—N2	−67.8 (2)	C6—C5—C10—C9	−32.9 (3)
S1—N1—N2—C10	173.9 (2)	C8—C9—C10—N2	−138.3 (3)
C6—C1—C2—C3	−2.2 (5)	C11—C9—C10—N2	38.4 (4)
C6—C1—C2—C17	177.4 (3)	C8—C9—C10—C5	31.5 (4)
C1—C2—C3—C4	3.3 (4)	C11—C9—C10—C5	−151.9 (3)
C17—C2—C3—C4	−176.4 (3)	C16—O3—C11—C12	−7.5 (5)
C18—O1—C4—C3	16.8 (4)	C16—O3—C11—C9	170.9 (3)
C18—O1—C4—C5	−162.3 (3)	C8—C9—C11—O3	−177.6 (3)
C2—C3—C4—O1	−178.6 (3)	C10—C9—C11—O3	5.7 (5)
C2—C3—C4—C5	0.5 (4)	C8—C9—C11—C12	0.9 (5)
O1—C4—C5—C6	174.1 (2)	C10—C9—C11—C12	−175.8 (3)
C3—C4—C5—C6	−5.1 (4)	O3—C11—C12—C13	178.4 (4)
O1—C4—C5—C10	−6.5 (4)	C9—C11—C12—C13	0.1 (5)
C3—C4—C5—C10	174.4 (3)	C11—C12—C13—C14	−0.3 (6)
C2—C1—C6—C5	−2.5 (5)	C11—C12—C13—C15	−178.4 (4)
C2—C1—C6—C7	175.3 (3)	C12—C13—C14—C8	−0.5 (5)
C4—C5—C6—C1	6.1 (4)	C15—C13—C14—C8	177.6 (4)
C10—C5—C6—C1	−173.4 (3)	C9—C8—C14—C13	1.4 (5)
C4—C5—C6—C7	−171.7 (3)	C7—C8—C14—C13	−177.7 (3)
C10—C5—C6—C7	8.8 (4)	O4—S1—C19—C24	−24.8 (3)
C1—C6—C7—O2	13.9 (5)	O5—S1—C19—C24	−157.5 (2)
C5—C6—C7—O2	−168.2 (3)	N1—S1—C19—C24	91.3 (3)
C1—C6—C7—C8	−160.9 (3)	O4—S1—C19—C20	157.0 (2)
C5—C6—C7—C8	17.0 (4)	O5—S1—C19—C20	24.3 (3)
O2—C7—C8—C14	−14.3 (5)	N1—S1—C19—C20	−86.9 (3)
C6—C7—C8—C14	160.5 (3)	C24—C19—C20—C21	0.5 (5)
O2—C7—C8—C9	166.6 (3)	S1—C19—C20—C21	178.6 (3)
C6—C7—C8—C9	−18.6 (4)	C19—C20—C21—C22	0.9 (5)

C14—C8—C9—C11	−1.6 (4)	C20—C21—C22—C23	−2.1 (5)
C7—C8—C9—C11	177.5 (3)	C20—C21—C22—C25	178.9 (4)
C14—C8—C9—C10	175.3 (3)	C21—C22—C23—C24	1.9 (5)
C7—C8—C9—C10	−5.6 (4)	C25—C22—C23—C24	−179.0 (3)
N1—N2—C10—C5	1.2 (4)	C22—C23—C24—C19	−0.6 (5)
N1—N2—C10—C9	169.5 (2)	C20—C19—C24—C23	−0.6 (5)
C4—C5—C10—N2	−44.2 (4)	S1—C19—C24—C23	−178.8 (2)

Hydrogen-bond geometry (Å, º)

D—H···*A*	*D*—H	H···*A*	*D*···*A*	*D*—H···*A*
C18—H18*C*···O4^{i}	0.96	2.62	3.441 (4)	143
C20—H20···O2^{ii}	0.93	2.62	3.511 (4)	161
N1—H1*A*···O1	0.85 (3)	1.92 (3)	2.680 (4)	148 (3)

Symmetry codes: (i) *x*+1, *y*, *z*; (ii) −*x*+1/2, −*y*+1, *z*+1/2.

Supplementary References

1. Omura, K. Rapid conversion of phenols to *p*-benzoquinones under acidic conditions with lead dioxide. *Synthesis* 1145–1148 (1998).
2. Savard, J. & Brassard, P. Regiospecific syntheses of quinones using vinylketene acetals derived from unsaturated esters. *Tetrahedron Lett.* **20**, 4911–4914 (1979).
3. Brady, R. M.; Hatzis, E.; Connor, T.; Street, I. P.; Baell, J. B. & Lessene, G. Synthesis of conformationally constrained benzoylureas as BH_3-mimetics. *Org. Biomol. Chem.* **10**, 5230–5237 (2012).
